# Supplementary material for: Magnetic Janus origami robot for cross-scale droplet omni-manipulation
Source: Nat Commun. 2023 Sep 6;14:5455. doi: 10.1038/s41467-023-41092-1 (PMC10482950; doi:10.1038/s41467-023-41092-1)
Supplement: Supplementary file 2 — Description of Additional Supplementary Files [file 41467_2023_41092_MOESM2_ESM.pdf]

Description of Additional Supplementary Files

Supplementary Movie Legends:

Supplementary Movie 1: Wrapping and transporting droplet by JO-robot.

Supplementary Movie 2: Three-dimensional droplet transportation.

Supplementary Movie 3: Versatile droplet manipulation based on JO-robot.

Supplementary Movie 4: Directional tumbling of JO-robot and JO-robot with droplet.

Supplementary Movie 5: Controllable daughter droplet dispensing.

Supplementary Movie 6: On-demand daughter droplet release.

Supplementary Movie 7: Dispensing of nanoliter droplet by mini JO-robot.

Supplementary Movie 8: On-demand microdroplets array generation.

Supplementary Movie 9: Droplet ejecting by JO-robot.

Supplementary Movie 10: Droplet mixing by the rotation of JO-robot and passive diffusion.

Supplementary Movie 11: Glycerol mixing by stirring and photothermal stirring of JO-robot.

Supplementary Movie 12: Integration of diverse droplet manipulation functions.

Supplementary Movie 13: Micro-sampling, sample transportation and addition, rapid reaction and products instant detection by JO-robot.

Supplementary Movie 14: Detachment of JO-robot.

Supplementary Movie 15: Versatile manipulation of nanoliter droplets.

Supplementary Movie 16: Nucleic acids extraction and purification.
