# Supplementary material for: Magnetic Janus origami robot for cross-scale droplet omni-manipulation
Source: Nat Commun. 2023 Sep 6;14:5455. doi: 10.1038/s41467-023-41092-1 (PMC10482950; doi:10.1038/s41467-023-41092-1)
Supplement: Supplementary file 3 — Supplementary Information [file 41467_2023_41092_MOESM3_ESM.pdf]

Supplementary Information

Magnetic Janus origami robot for cross-scale droplet omni-manipulation.

Shaojun Jiang[1, 2], Bo Li[3], Jun Zhao[4], Dong Wu[1], Yiyuan Zhang[2], Zhipeng Zhao[2], Yiyuan Zhang[1], Hao Yu[3], Kexiang Shao[1], Cong Zhang[1], Rui Li[1], Chao Chen[1], Zuojun Shen[5], Jie Hu[5], Bin Dong[1], Ling Zhu[4], Jiawen Li[1], Liqiu Wang[6]*, Jiaru Chu[1], Yanlei Hu[1]**

[1]CAS Key Laboratory of Mechanical Behavior and Design of Materials, Department of Precision Machinery and Precision Instrumentation, University of Science and Technology of China, Hefei, Anhui 230027, China
[2]Department of Mechanical Engineering, The University of Hong Kong, Hong Kong, China
[3]CAS Key Laboratory of Mechanical Behavior and Design of Materials, Department of Modern Mechanics, University of Science and Technology of China, Hefei 230027, China
[4]Center of Engineering Technology Research for Biomedical Optical Instrument, Hefei Institutes of Physical Science, Chinese Academy of Sciences, Hefei, Anhui 230031, China
[5]Department of Clinical Laboratory, The First Affiliated Hospital of USTC, Division of Life Sciences and Medicine, University of Science and Technology of China, Hefei, Anhui 230001, China
[6]Department of Mechanical Engineering, The Hong Kong Polytechnic University, Hong Kong, China.

*Corresponding author.
Email: dongwu@ustc.edu.cn (D. W.); liqiu.wang@polyu.edu.hk (L. W.); huyl@ustc.edu.cn (Y. H.)

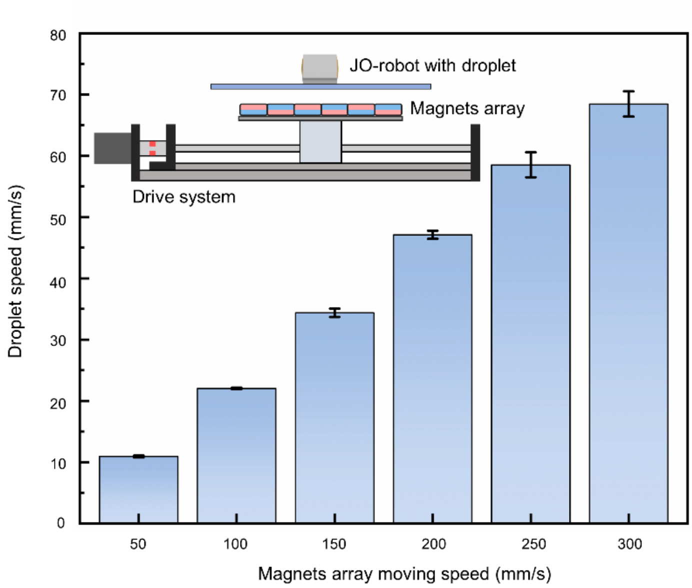

Supplementary Figure 1. Droplet transport speed versus magnets array moving speed. The JO-robot is driven by the horizontal movement of the magnets array. The droplet transport speed increases with the moving speed of the magnets array and it can reach 68.47 ± 2.1 mm/s (6.5 μL droplet, corresponding to the magnets array moving speed of 300 mm/s). Further increasing the magnets array moving speed may cause some of the liquid wrapped in the JO-robot to be thrown out during the high-speed transportation, resulting in leakage. As the droplet volume decreases, the droplet transport speed can be further increased. For example, the transport speed of a 4 μL droplet can reach 78.84 ± 3.4 mm/s with the magnets array moving speed of 360 mm/s. The error bar represents the standard deviation of five independent measurements. Source data are provided as a Source Data file.

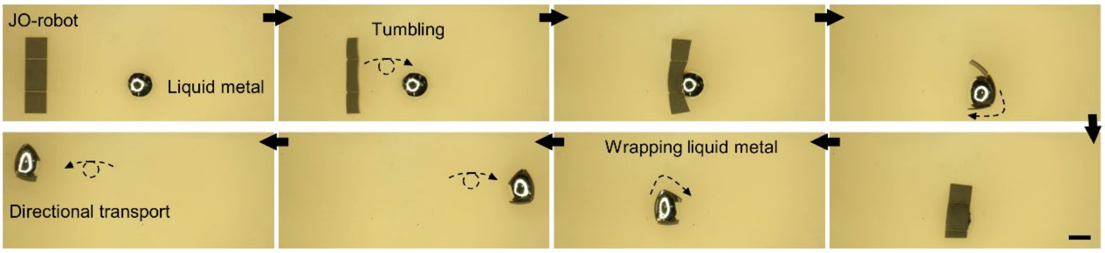

Supplementary Figure 2. Liquid metal manipulation by JO-robot. The JO-robot actively approaches the liquid metal (gallium-indium-tin alloys) droplet by tumbling. The unmodified surface of the JO-robot comes into contact with the liquid metal and wraps the liquid metal under the action of the surface oxide layer. The laser-modified surface has rough micro/nanostructures, which gives it low adhesion to liquid metal and cannot be adhered by the liquid metal, while the unmodified surface has high adhesion to the liquid metal. Therefore, the Janus characteristic of JO-robot is still effective for liquid metal. After wrapping the liquid metal, JO-robot can carry the liquid metal on the microstructure surface for untethered transportation. Scale bar is 2 mm.

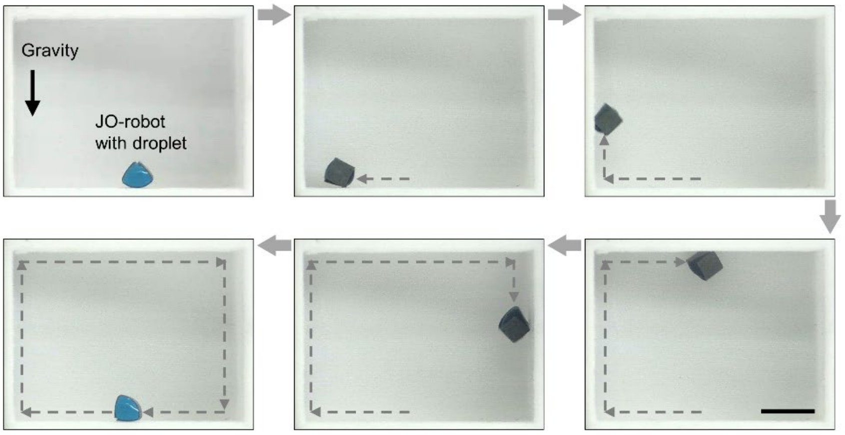

Supplementary Figure 3. Three-dimensional droplet manipulation by JO-robot. With good magnetic response characteristic, JO-robot can transport droplet in three-dimensional space. By using a rotating cube permanent magnet (25 mm × 25 mm × 25 mm), the JO-robot can carry the droplet (6.5 μL) to tumble along the horizontal and vertical walls. It can climb against gravity on the vertical wall, transport on the inverted surface, and transport downward along gravity on the vertical wall, showing a good three-dimensional droplet transport capacity. Scale bar is 5 mm.

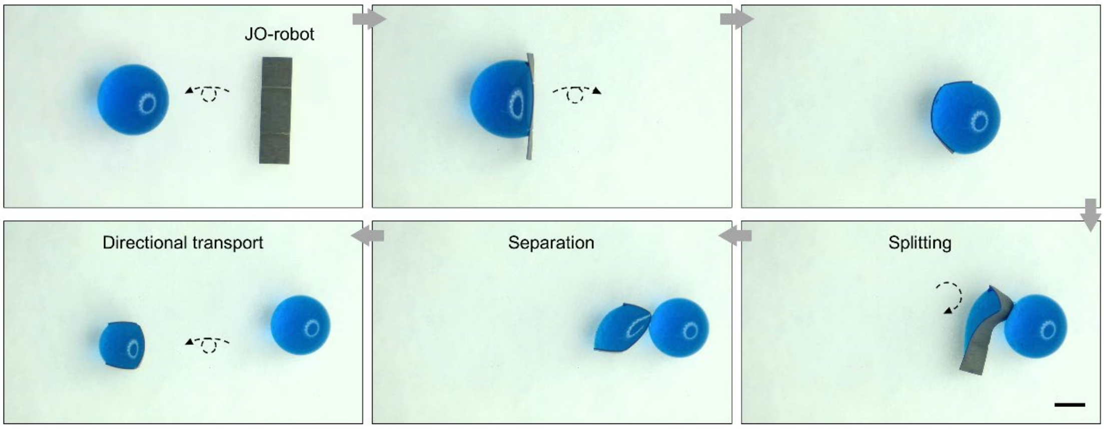

Supplementary Figure 4. Droplet splitting by JO-robot. In addition to daughter droplet dispensing, JO-robot can also realize droplet splitting. First, JO-robot approaches the free droplet on the superhydrophobic substrate by tumbling. Upon touching the droplet, the hydrophobic side of the JO-robot can spontaneously stick to the droplet surface. And then, JO-robot splits a part of liquid from the droplet by tumbling, and separates it from the large droplet by using the superhydrophobic outer surface. Finally, JO-robot transports the droplet by tumbling. Scale bar is 2 mm.

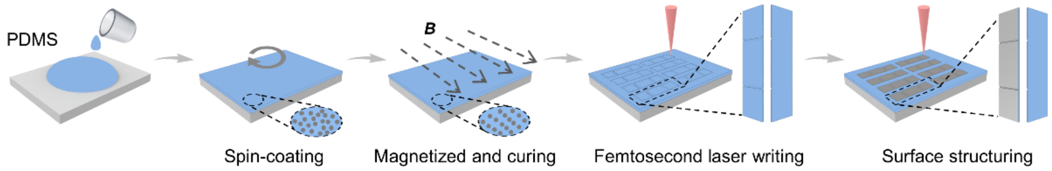

Supplementary Figure 5. Schematic illustration of the fabrication procedure of JO-robot. Carbonyl iron particles doped polydimethylsiloxane (PDMS) is first poured onto the glass substrate and then spin coated. The iron particles inside the PDMS are aligned into orientated chains by applying a directional magnetic field. After thermal curing, a solidified magnetic film (95.9 μm ± 3.5 μm) with carbonyl iron particles chains can be obtained. In femtosecond laser processing, the contour cutting, creases scanning and wettability modification of the JO-robot can be achieved in a single processing step by importing the designed scanning paths into the software, setting the path processing sequence and processing speed. The contour and creases of the JO-robot are fabricated by femtosecond laser direct writing. By controlling the laser writing direction, the carbonyl iron particles chains are perpendicular to the long side of the JO-robot. And then, the top surface of the JO-robot is modified in-situ by femtosecond laser by sequentially loading the scanning path and scanning speed. After laser modification, the top surface of the JO-robot becomes superhydrophobic with low droplet adhesion, while the unmodified side (bottom side) is hydrophobic with high droplet adhesion. By carefully removing the processed sample from the glass substrate, JO-robot can be successfully obtained. This laser in-situ processing method enables rapid batch manufacturing of JO-robot.

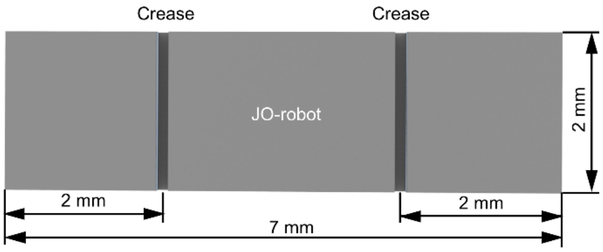

Supplementary Figure 6. The detailed size of JO-robot. The size of JO-robot is 2 mm × 7 mm. There are two creases on the superhydrophobic surface. The distance between the center of the crease and the short side of JO-robot is 2 mm. Based on this design size, JO-robot can form a closed triangular section when folded along the crease.

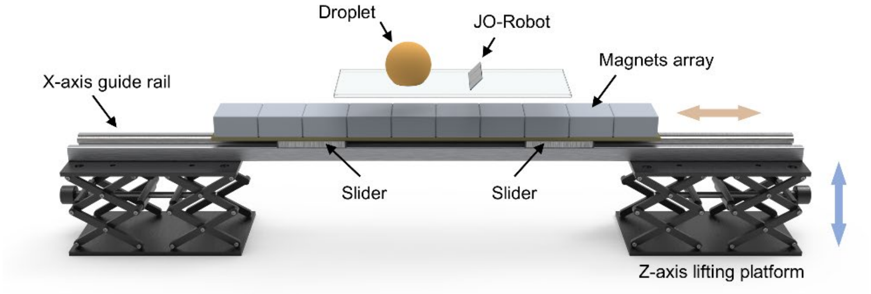

Supplementary Figure 7. Schematic diagram of the magnetic drive system. The JO-robot is driven by the magnets array. The distance between the magnets array and JO-robot can be adjusted by the Z-axis lifting platform to control the magnetic flux density. The magnets array is carried by the sliders and moves horizontally on the X-axis guide rail to drive the JO-robot for droplet manipulation.

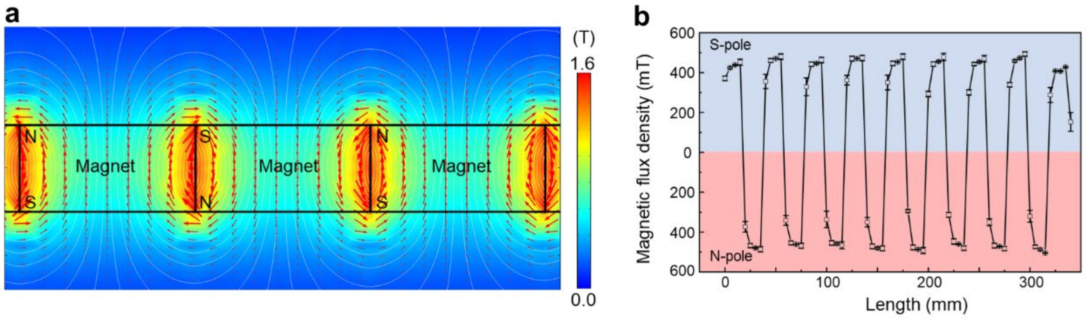

Supplementary Figure 8. Magnetic field simulation and measured magnetic flux density of the magnets array. (a) The periodic magnetic field generated by the magnets array. A series of rectangular permanent magnets (20 mm × 20 mm × 10 mm) are attracted to each other end-to-end. And the N-pole and S-pole are distributed up and down. The simulation shows the magnetic field generated by three magnets selected in the magnets array. It can be seen that the magnetic field is distributed periodically in space. (b) The measured magnetic flux density perpendicular to the surface of the magnets array. Here, seventeen permanent magnets are used to form the magnets array. It shows that the magnetic flux density on the magnets array is distributed periodically. The error bar represents the standard deviation of five independent measurements. Source data are provided as a Source Data file.

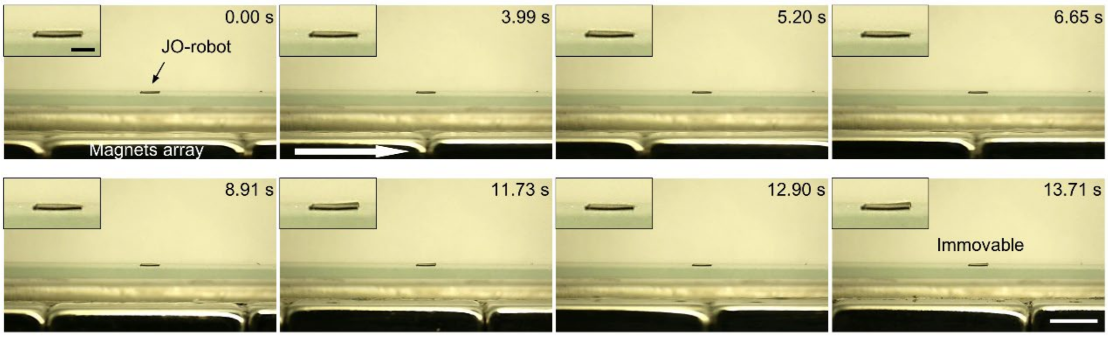

Supplementary Figure 9. Magnetic response state of the JO-robot with uniformly dispersed carbonyl iron particles. As the magnets array moves to the right, the JO-robot with uniformly dispersed carbonyl iron particles (without oriented carbonyl iron particles chains) cannot be driven by the magnets array. Scale bar is 5 mm. The inset is the enlarged view of JO-robot. Scale bar is 1mm.

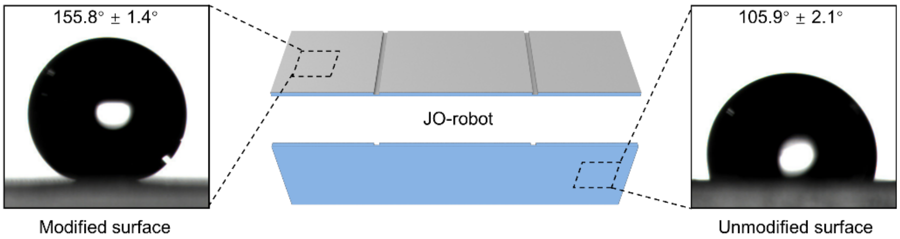

Supplementary Figure 10. Water contact angle of the JO-robot. JO-robot has the Janus characteristic. The surface modified by femtosecond laser is superhydrophobic with the water contact angle of 155.8° ± 1.4°, while the unmodified surface is hydrophobic with the water contact angle of 105.9° ± 2.1°.

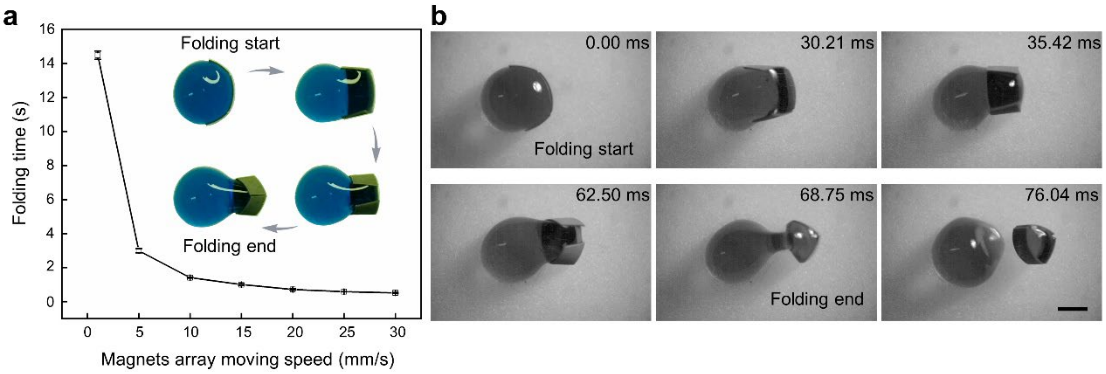

Supplementary Figure 11. The folding time of the JO-robot. (a) Dependence of the folding time of the JO-robot on the magnets array moving speed. As the magnets array moving speed increases from 0 to 30 mm/s, the folding time of the JO-robot decreases from 14.48 s ± 0.25 s to 0.52 s ± 0.03 s. The inset shows the entire folding process of the JO-robot. (b) The high-speed folding of JO-robot. The folding of the JO-robot can be realized in 68.75 ms. Scale bar is 2 mm. The error bar represents the standard deviation of five independent measurements. Source data are provided as a Source Data file.

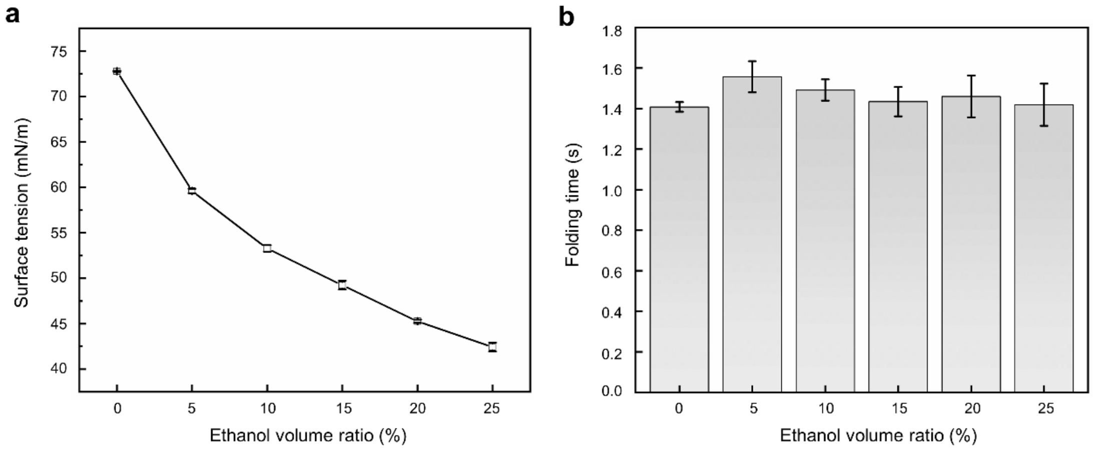

Supplementary Figure 12. Influence of liquid surface tension on folding time of JO-robot. (a) Surface tension of ethanol solutions with different volume ratios (the ratio of the volume of ethanol to the volume of water). When the volume ratio of ethanol to water increased from 0 to 25%, the liquid surface tension decreased rapidly from 72.7 mN/m to 42.4 mN/m. (b) Dependence of the folding time of the JO-robot on ethanol solutions with different volume ratios. The liquid surface tension has little effect on the folding time of the JO-robot. The folding time is between 1.42 s and 1.56 s when the moving speed of magnets array is 10 mm/s. The error bar represents the standard deviation of five independent measurements. Source data are provided as a Source Data file.

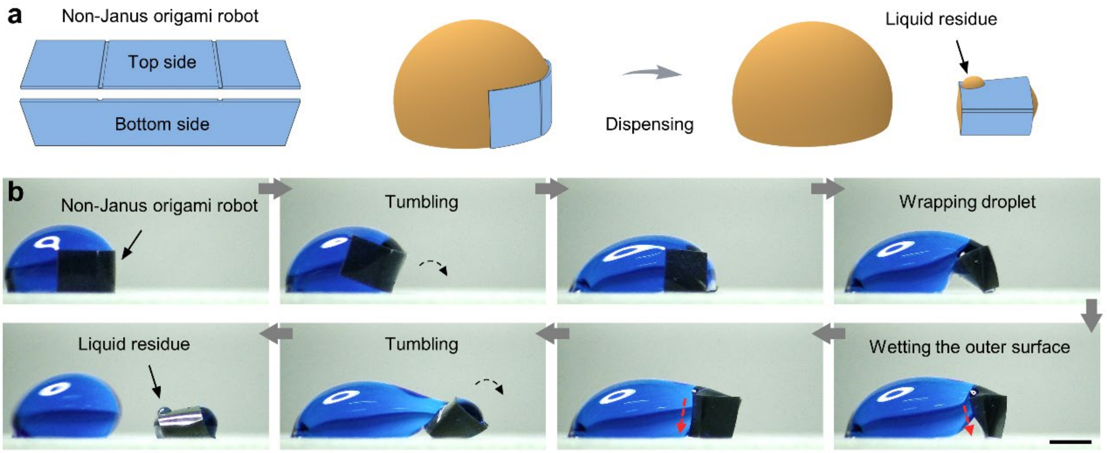

Supplementary Figure 13. Daughter droplet dispensing by the origami robot without Janus characteristic (non-Janus origami robot). (a) Schematic diagram of the non-Janus origami robot (both two sides are hydrophobic with high droplet adhesion) and the process of dispensing daughter droplet by non-Janus origami robot. When the non-Janus origami robot dispenses droplet, there will be liquid residue on the outer surface. (b) Daughter droplet dispensing by non-Janus origami robot. Initially, the non-Janus origami robot wraps the droplet like the JO-robot. However, when the non-Janus origami robot tumbles further to the right under the action of the magnetic field, the droplet wets the outer surface of the robot. There is no necking point at the connection between the large droplet and the robot. Different from the droplet separation caused by the breakage of the necking point when JO-robot dispenses daughter droplet, the liquid slides on the outer surface of the non-Janus origami robot, and finally separates from the outer surface. Due to the high adhesion of the outer surface of the non-Janus origami robot, some liquid can remain on the outer surface. Scale bar is 2 mm.

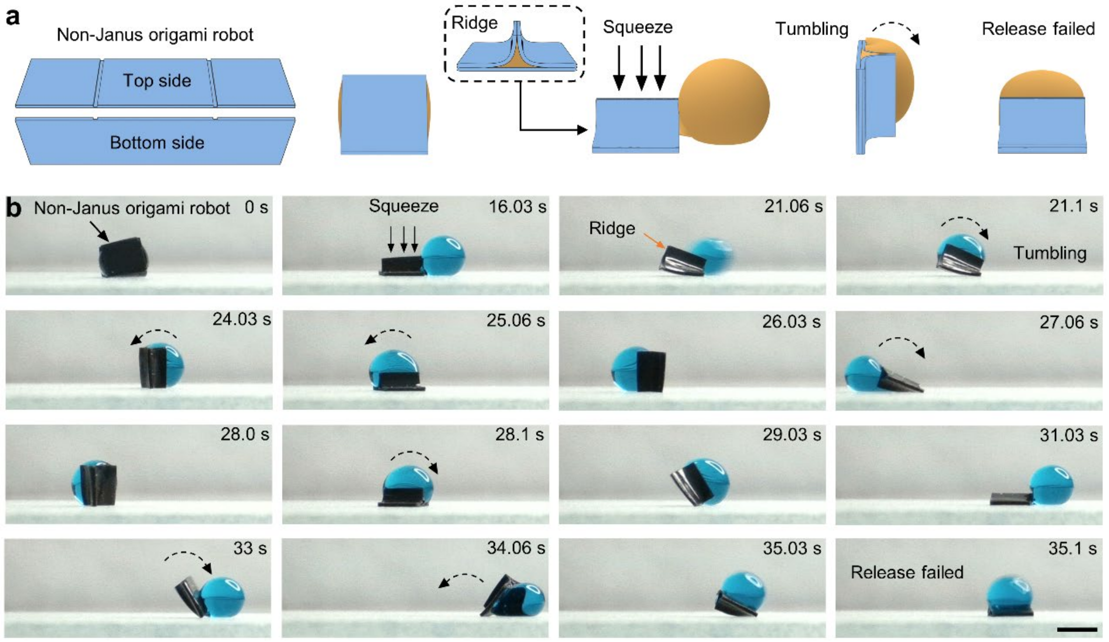

Supplementary Figure 14. Daughter droplet release by the non-Janus origami robot. (a) Schematic diagram of the non-Janus origami robot and the process of releasing daughter droplet by non-Janus origami robot. (b) Optical images of the process of releasing daughter droplet by non-Janus origami robot. The non-Janus origami robot is first kept slightly tilted to facilitate the directional extrusion of the droplet. Due to the non-Janus feature, the left and right sides of the non-Janus origami robot can interfere with each other during the folding deformation, resulting in the formation of a raised ridge. Some liquid remains in this ridge, and is difficult to be completely squeezed out. Subsequently, when the non-Janus origami robot is controlled to tumble, the daughter droplet sticks to the outer surface of the robot and cannot be released (the outer surface of the non-Janus origami robot is hydrophobic with high droplet adhesion). Scale bar is 5 mm.

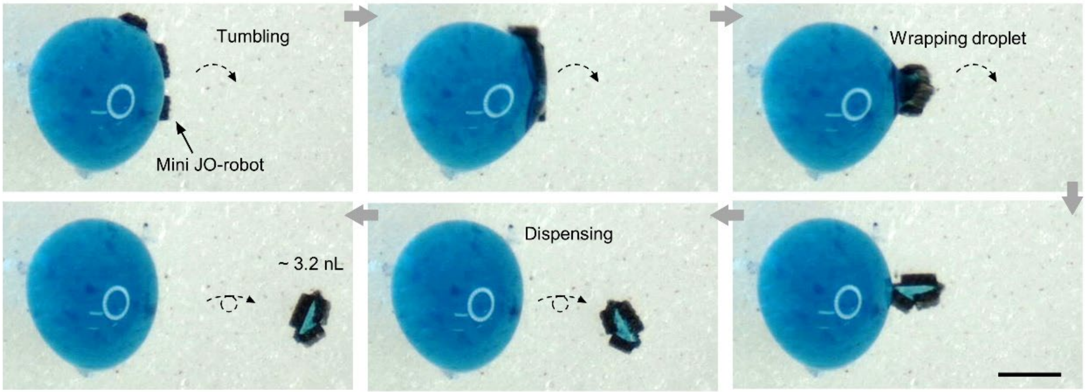

Supplementary Figure 15. Dispensing of nanoliter droplet by mini JO-robot. JO-robot has stable droplet manipulation capability and can realize cross-scale droplet manipulation. Based on the flexible femtosecond laser micro/nano fabrication technology, mini JO-robot (230 μm × 940 μm) can be easily fabricated to realize controllable and high-precision dispensing of nanoliter daughter droplet. Similar to the large JO-robot, the mini JO-robot folds along the creases and wraps part of the liquid under the combined action of magnetic field and capillary force. Droplet with volume of ~3.2 nL can be dispensed and transported directionally by the mini JO-robot. Scale bar is 500 μm.

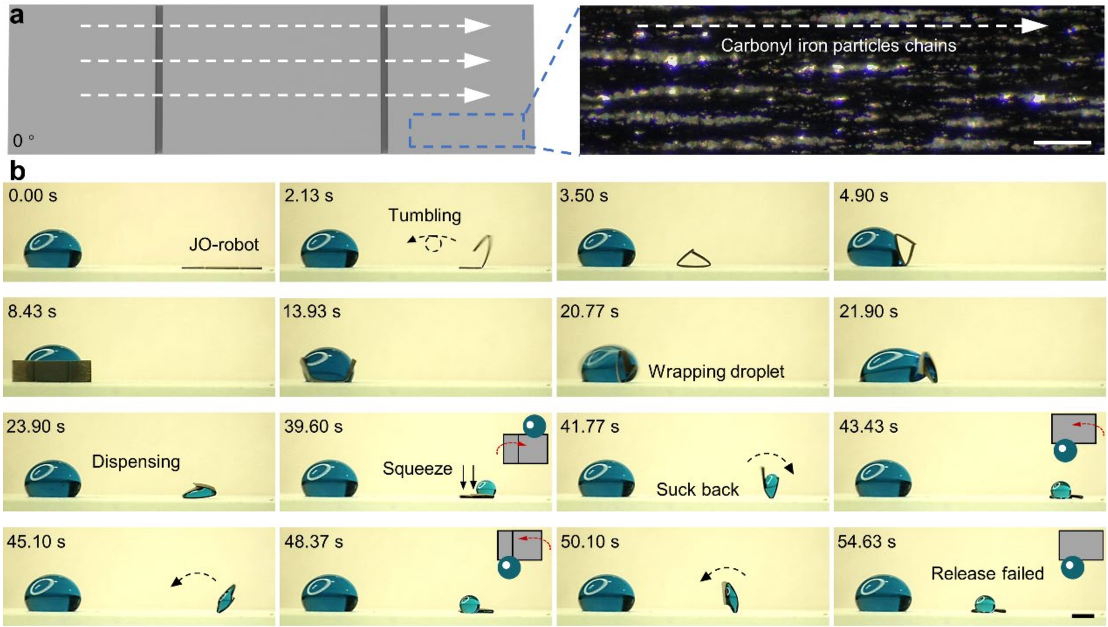

Supplementary Figure 16. Droplet manipulation by JO-robot with magnetic particles aligned along the long side (JO-robot (0°)). (a) Schematic diagram of JO-robot with carbonyl iron particles aligned along the long side (i.e., the angle between the chains and the long side is 0°). The dotted arrow indicates the orientation of the carbonyl iron particles chains. Optical image shows the carbonyl iron particles chains inside the JO-robot (0°). Scale bar is 100 µm. (b) Droplet manipulation by JO-robot (0°). The JO-robot (0°) tumbles around the short side to approach and touch the droplet. Since the tumbling axis is the short side, the JO-robot (0°) folds along the creases during the tumbling. Some of the liquid can be wrapped and dispensed by the JO-robot (0°) through the controlled tumbling. By increasing the magnetic field strength, the wrapped liquid can be squeezed out. However, when the JO-robot (0°) tries to release the droplet by tumbling, the droplet is sucked back into the robot, and cannot be released by the JO-robot (0°). This is because the squeezed droplet is located on the side of the robot (relative to the tumbling direction) and it cannot be pushed away by the JO-robot with the superhydrophobic surface. The insets show the relative position of the squeezed droplet and the JO-robot. Scale bar is 2 mm.

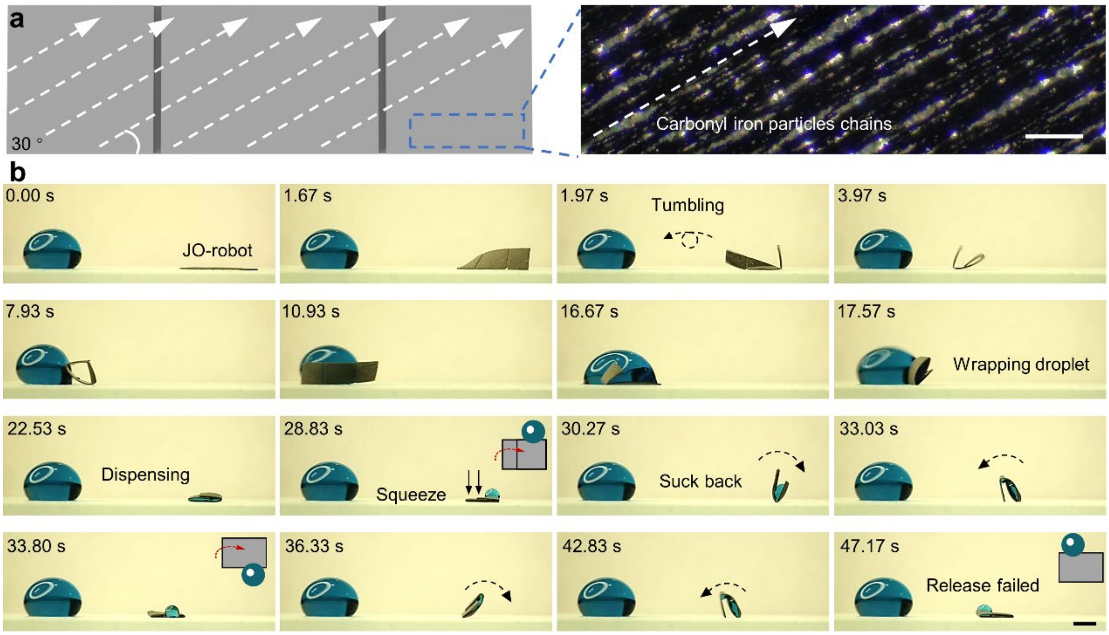

Supplementary Figure 17. Droplet manipulation by JO-robot with an angle of 30 degrees between the magnetic particles chains and the long side (JO-robot (30°)). (a) Schematic diagram of JO-robot (30°). The dotted arrow indicates the orientation of the carbonyl iron particles chains. Optical image shows the carbonyl iron particles chains inside the JO-robot (30°). Scale bar is 100 μm. (b) Droplet manipulation by JO-robot (30°). The JO-robot (30°) folds along the creases and tumbles around the short side. After contacting the water droplet, JO-robot (30°) can dispense droplet by tumbling and wrapping part of the liquid. Similar to JO-robot (0°), JO-robot (30°) is also difficult to release the dispensed daughter droplet. The insets show the relative position of the squeezed droplet and the JO-robot. The squeezed droplet is located on the side of the JO-robot. Scale bar is 2 mm.

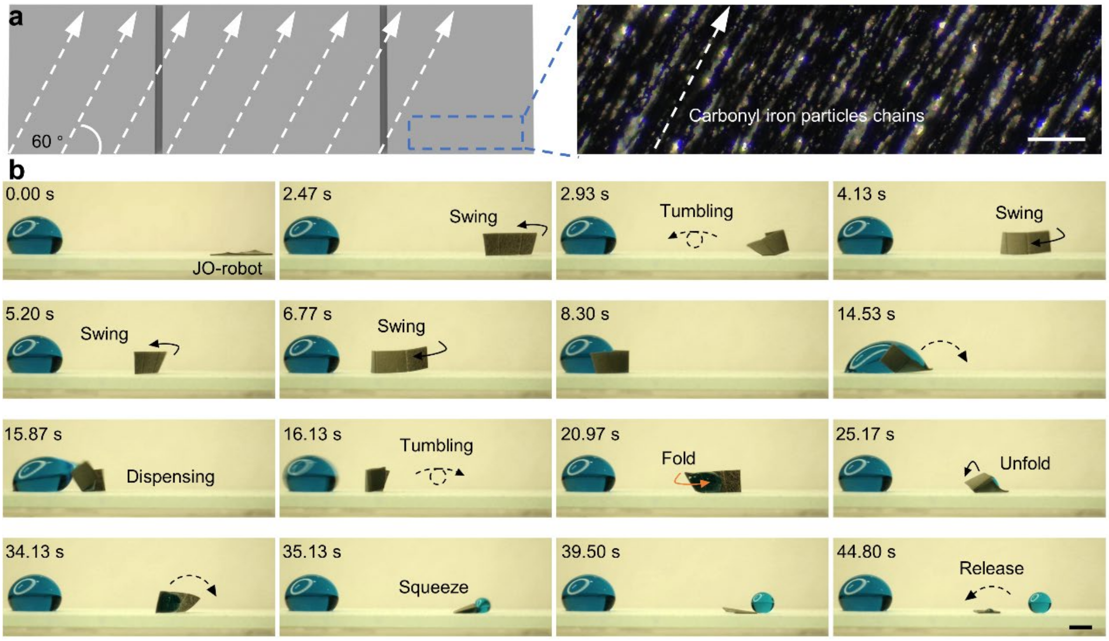

Supplementary Figure 18. Droplet manipulation by JO-robot with an angle of 60 degrees between the magnetic particles chains and the long side (JO-robot (60°)). (a) Schematic diagram of JO-robot (60°). The dotted arrow indicates the orientation of the carbonyl iron particles chains. Optical image shows the carbonyl iron particles chains inside the JO-robot (60°). Scale bar is 100 μm. (b) Droplet manipulation by JO-robot (60°). The JO-robot (60°) tumbles around the long side. However, the movement of JO-robot (60°) is very unstable. The robot swings repeatedly during the movement. The unstable swing of the robot originates from the misalignment between the tumbling axis and the long side. The tumbling axis is perpendicular to the carbonyl iron particles chains, so the angle between the tumbling axis and the long side is 30 degrees. After wrapping the droplet, the JO-robot (60°) can also dispense the daughter droplet by tumbling. To release the droplet, the JO-robot (60°) turns the side carrying the droplet downward. When the magnetic field increases, the droplet between the robot and the superhydrophobic substrate can be squeezed out. After that, the JO-robot (60°) tumbles to detach the droplet for daughter droplet release. Although daughter droplet dispensing and release can be realized, the controllability of the tumbling process and droplet manipulation process of the JO-robot (60°) is poor. Scale bar is 2 mm.

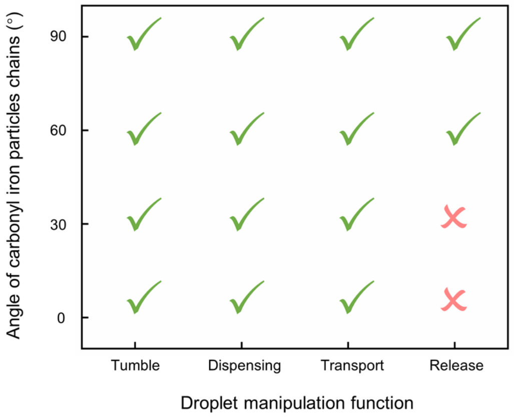

Supplementary Figure 19. The droplet manipulation functions that can be realized by JO-robot with different carbonyl iron particles chains angles. The green hook means that the function can be realized. And the red cross means that the function cannot be realized.

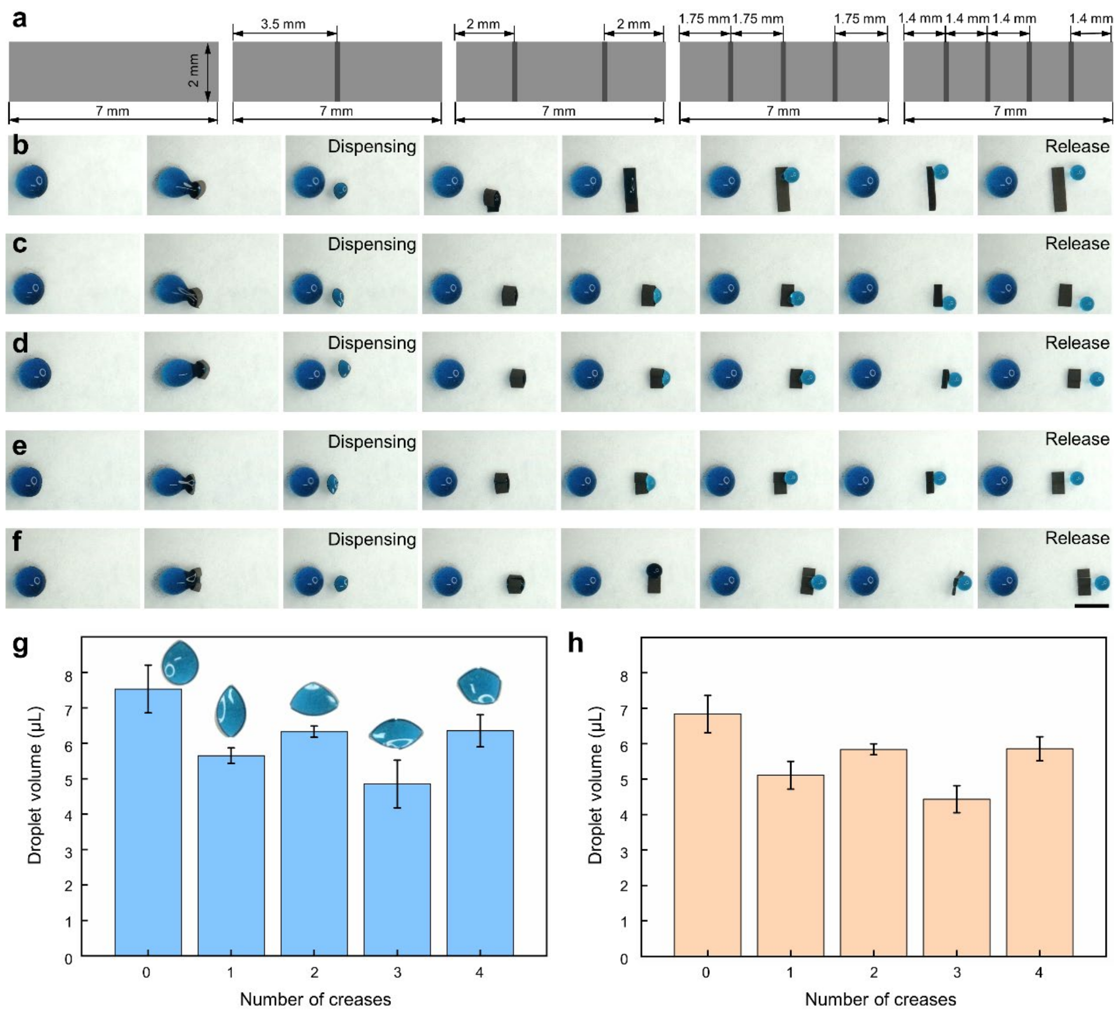

Supplementary Figure 20. Daughter droplet dispensing and release by JO-robots with different number of creases. (a) The schematic diagram of the JO-robots with different number of creases. (b-f) JO-robots with 0 to 4 creases are used for daughter droplet dispensing and release, respectively. The JO-robots with different number of creases can dispense daughter droplet by folding along the creases. By increasing the magnetic field strength, JO-robots can squeeze out daughter droplets and release the droplets by tumbling and pushing. Among them, it is worth mentioning that the dispensing and release processes are slightly different for the JO-robot with no crease (NC-JO-robot). When dispensing daughter droplet, the NC-JO-robot bends along the central axis to wrap the liquid and detaches the droplet by tumbling. When NC-JO-robot folds to release the daughter droplet, due to the elasticity of the robot, there is always a gap in the middle part of the folded NC-JO-robot. The droplet is difficult to be completely squeezed out, resulting in the failure of the release. In order to release the daughter droplet, the NC-JO-robot first unfolds under the control of the magnetic field, and then squeezes out the daughter droplet by interaction with the substrate. The NC-JO-robot can eventually realize daughter droplet release by using the superhydrophobic outer surface to gently push the droplet. Scale bar is 5 mm. (g, h) Volume of daughter droplets that can be (g) dispensed and (h) released by JO-robots with different creases. It shows that the JO-robots with 1 and 3 creases dispense slightly smaller droplets than other

robots. The NC-JO-robot can dispense the largest droplet, but its operation is complicated as described above. Therefore, the JO-robot with two creases can meet the requirements of processing efficiency, manipulation convenience and droplet manipulation performance. The insets show the top view of the JO-robots wrapping water droplets. The error bar represents the standard deviation of five independent measurements. Source data are provided as a Source Data file.

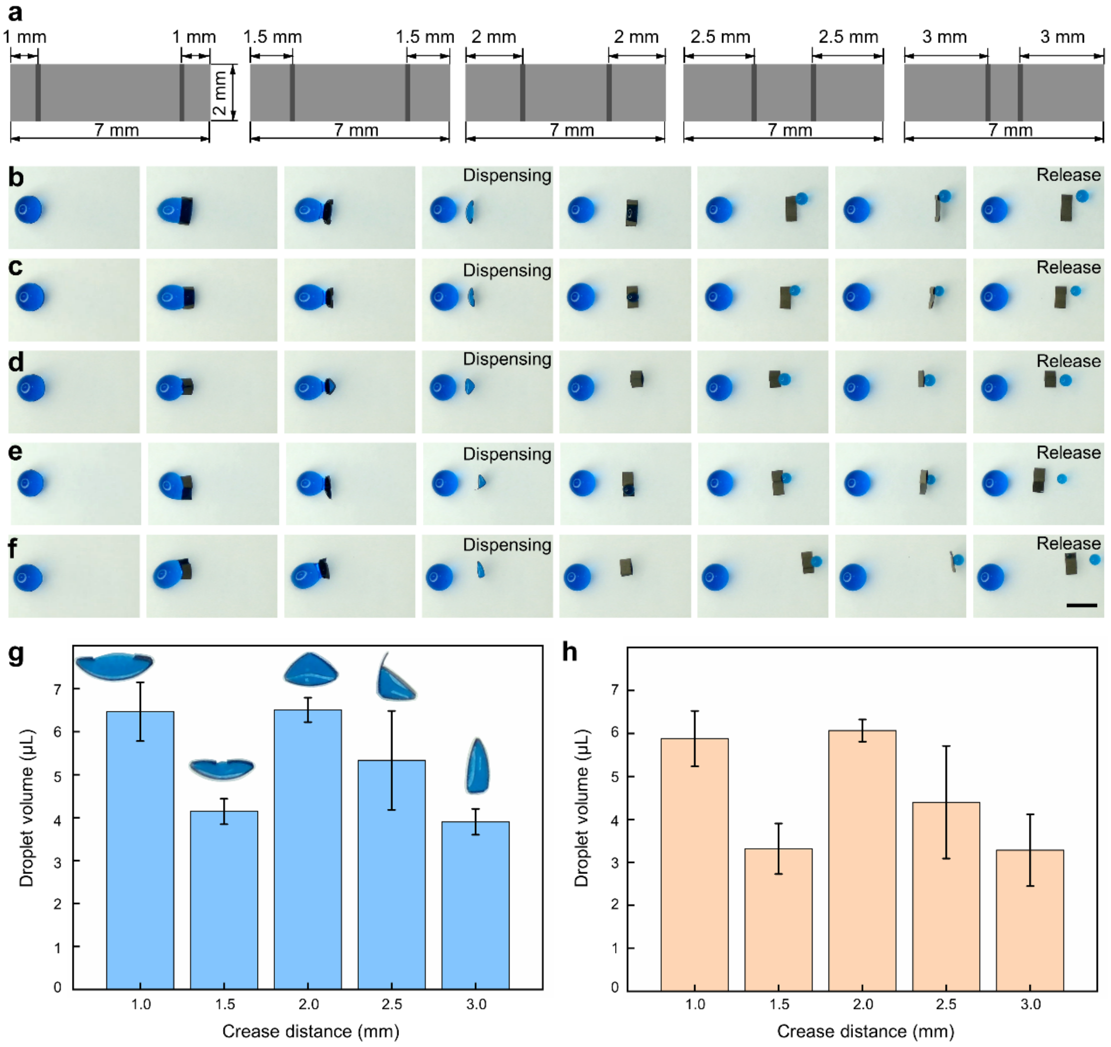

Supplementary Figure 21. Daughter droplet dispensing and release by JO-robots with different crease distributions. (a) The schematic diagram of the JO-robots with different crease distributions. (b-f) JO-robots with the distance between the crease and the short side from 1 mm to 3 mm are used for daughter droplet dispensing and release, respectively. Scale bar is 5 mm. When the middle plate is longer than the side plates (JO-robot can be seen as three plates connected by two creases), the plates on both sides can fold symmetrically toward the middle plate when dispensing droplets (Figure b-d). When the crease distances are 1 mm and 1.5 mm, the JO-robots cannot fold to form the closed triangles. However, due to the origami deformation ability and the Janus wetting characteristic, they can still dispense and release droplets (Figure b, c). When the length of the two side plates is longer than the middle plate, the JO-robot tends to fold asymmetrically along the side plates when dispensing droplets, making the dispensing process less controllable (Figure e, f). (g, h) Volume of daughter droplets that can be (f) dispensed and (g) released by JO-robots with different crease distances. The insets show the top view of the JO-robots wrapping water droplets. The error bar represents the standard deviation of five independent measurements. Source data are provided as a Source Data file.

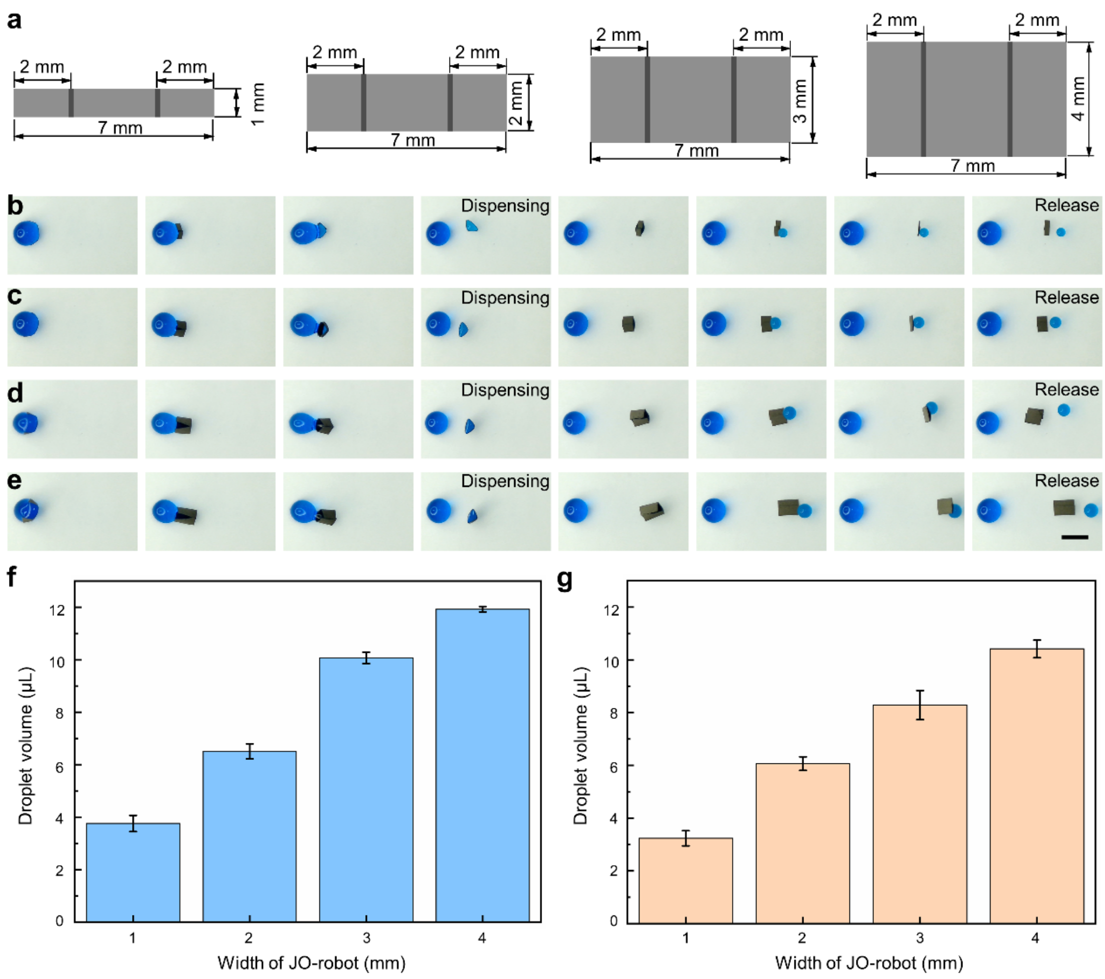

Supplementary Figure 22. Daughter droplet dispensing and release by JO-robots with different widths. (a) The schematic diagram of the JO-robots with different widths. (b-e) JO-robots with widths from 1 mm to 4 mm are used for daughter droplet dispensing and release, respectively. Scale bar is 5 mm. The JO-robots with different widths can realize the droplet dispensing and release. (f, g) Volume of daughter droplets that can be (f) dispensed and (g) released by JO-robots with different widths. The volume of dispensed droplets increases with the width of the robot. However, the volume of residual liquid also increases with the width of the robot. The error bar represents the standard deviation of five independent measurements. Source data are provided as a Source Data file.

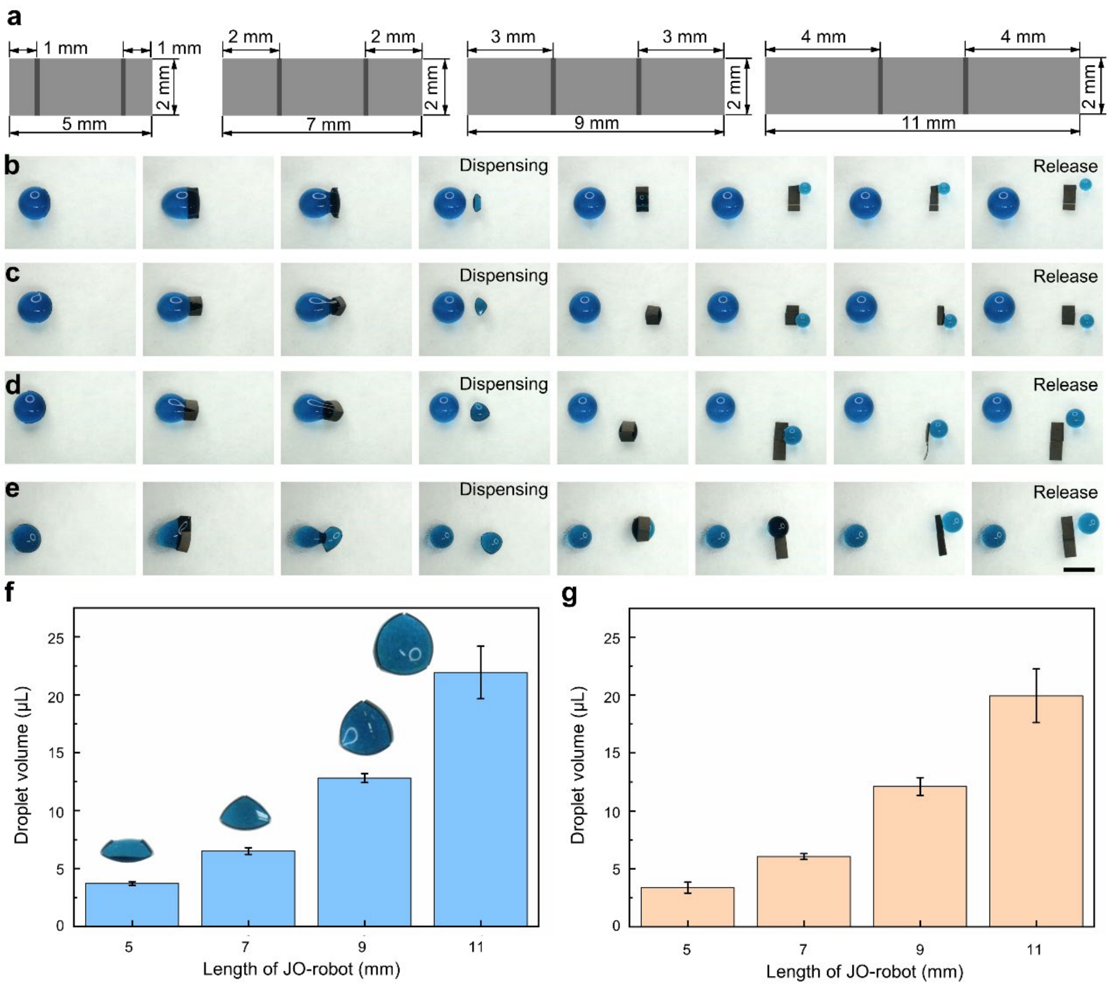

Supplementary Figure 23. Daughter droplet dispensing and release by JO-robots with different lengths. (a) The schematic diagram of the JO-robots with different lengths. (b-e) JO-robots with lengths from 5 mm to 11 mm are used for daughter droplet dispensing and release, respectively. When the length of the side plate is 1 mm, the JO-robot cannot fold to form a closed triangle (Figure b). During dispensing process, the plates on both sides folds symmetrically toward the middle plate when the middle plate is longer than the side plates (Figure b-d). However, the JO-robot tends to fold asymmetrically along the side plates when the length of the middle plate is shorter than the side plates, making the dispensing process less controllable (Figure e). Scale bar is 5 mm. (f, g) Volume of daughter droplets that can be (f) dispensed and (g) released by JO-robots with different widths. The volume of dispensed droplets increases with the length of the robot. The insets show the top view of the JO-robots wrapping water droplets. The error bar represents the standard deviation of five independent measurements. Source data are provided as a Source Data file.

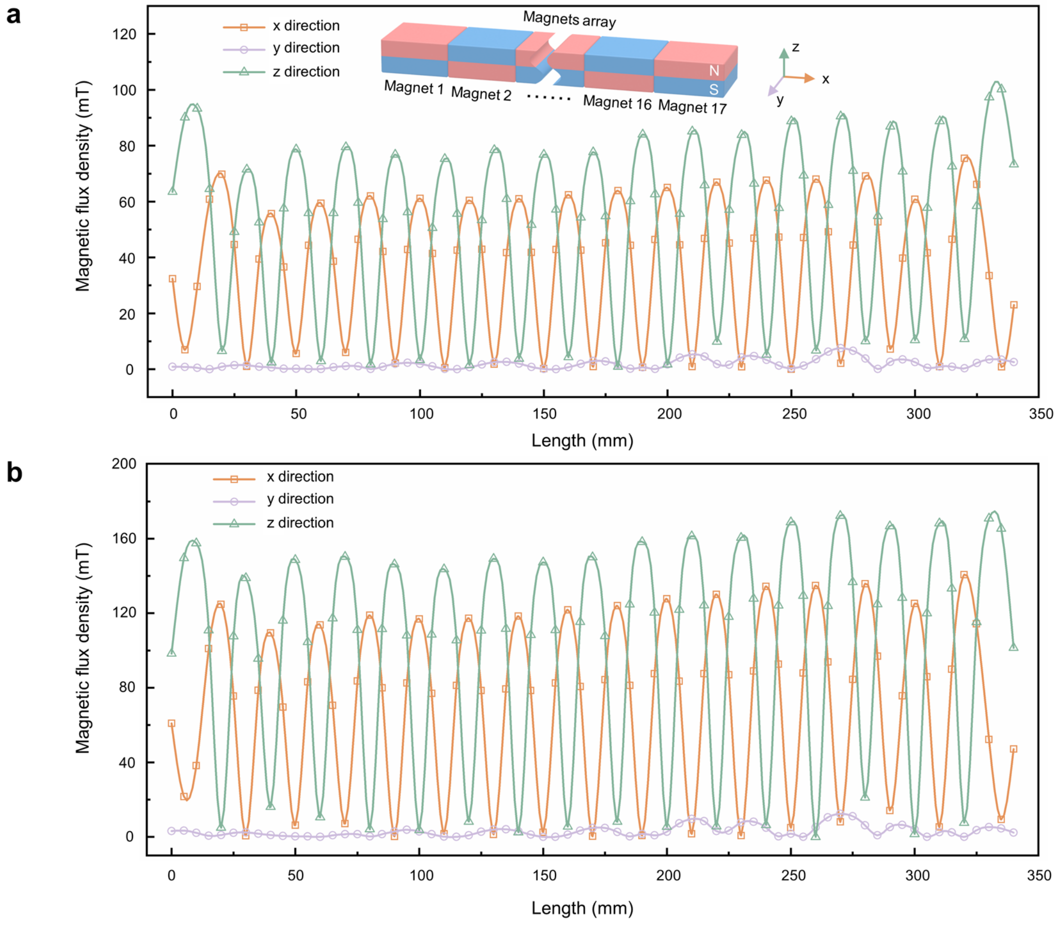

Supplementary Figure 24. The magnetic flux density (x-, y- and z-directions) that enables the droplet manipulation functions including the tumbling of the JO-robot, the dispensing and transportation of droplet. (a) Minimum magnetic flux density for droplet manipulation. The corresponding distance between the JO-robot and the upper surface of the magnets array is ~12 mm. The inset shows the schematic of the magnets array. (b) Maximum magnetic flux density for droplet manipulation. The corresponding distance between the JO-robot and the upper surface of the magnets array is ~8 mm. The length in the horizontal coordinate refers to the length of the magnet array. For the convenience of description, the average value of the local maximum magnetic flux density in the z-direction of the working space of JO-robot (at the position where the magnetic flux density is nearly zero in both the x- and y- directions) is simply used to describe the magnetic flux density information. The main droplet manipulation processes are carried out in the region away from both ends of the magnets array, so the statistical range of the magnetic flux density in calculating the average value is between the positions at 30 mm and 310 mm of the magnets array. So, the magnetic flux densities in (a) and (b) are simply described by 81.5±5.7 mT and 155.4±10.4 mT, respectively. Source data are provided as a Source Data file.

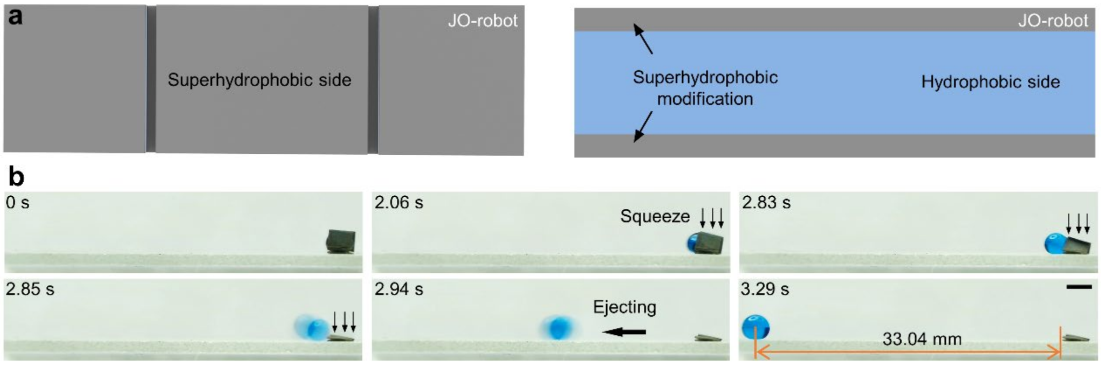

Supplementary Figure 25. Droplet ejecting by JO-robot. (a) The edges on both sides of the hydrophobic surface of the JO-robot are modified by the femtosecond laser to transform them into superhydrophobic and low adhesion state. The width of the modified superhydrophobic area is ~500 um. The further modification of the hydrophobic surface reduces its adhesion to the droplet. (b) The modified JO-robot can realize droplet ejection. By increasing the magnetic field strength, JO-robot can be attracted downward to squeeze the droplet wrapped in it (~6.5 μL). With the increase of the magnetic field strength, the droplet is eventually ejected under the squeeze of JO-robot. The average ejection speed reaches ~75.09 mm/s and the ejection distance is ~33.04 mm. Scale bar is 2 mm.

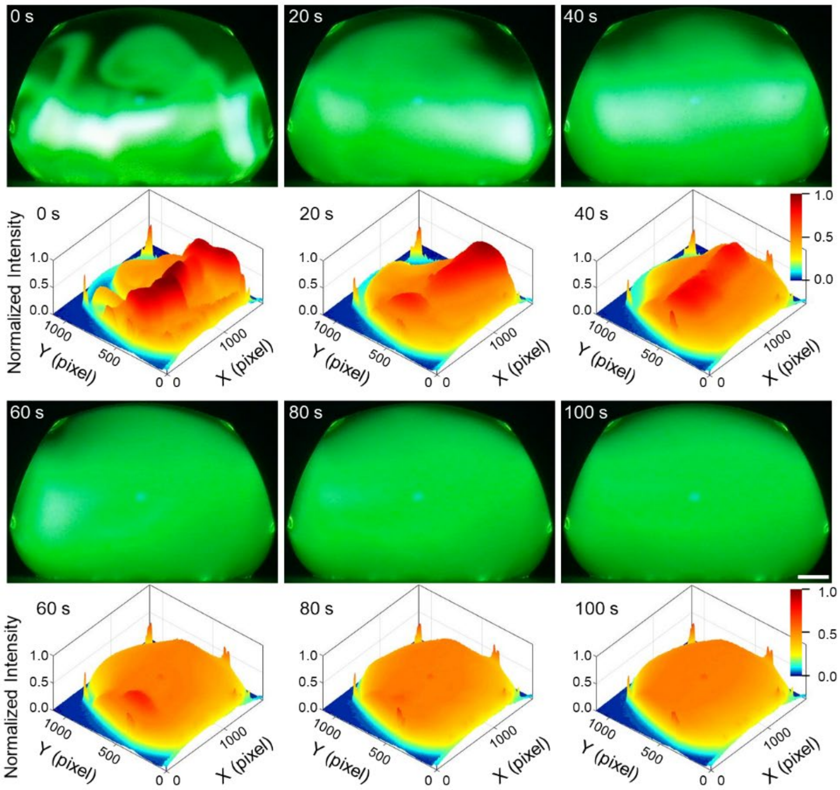

Supplementary Figure 26. Droplet mixing by passive diffusion. The water droplet (6.5 μL) containing 0.01 M fluorescein sodium salt is added to the sessile droplet (30 μL) to monitor the mixing process. In order to compare the mixing effect, JO-robot is also suspended on the droplet surface. It can be seen that the fluorescence is gradually distributed to the whole droplet with the passive diffusion of the liquid. The normalized two-dimensional fluorescence distribution maps show that the fluorescence intensity distribution inside the droplet gradually tends to be uniform with the passive diffusion of the liquid. The droplet achieves uniform mixing at 100 seconds. The active uniform mixing achieved by JO-robot rotation takes only 5 seconds, which proves the contribution of JO-robot to the rapid mixing of liquids. Scale bar is 500 μm.

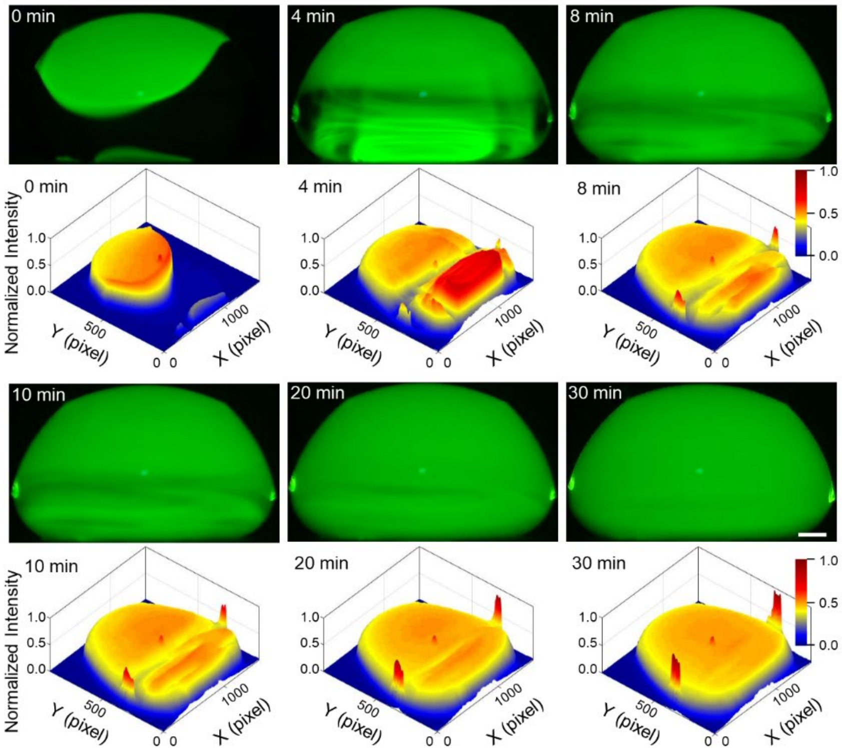

Supplementary Figure 27. Stirring glycerol droplet by JO-robot without remote heating. The glycerol droplet (6.5 μL) containing 0.01 M fluorescein sodium salt is added to the sessile glycerol droplet (30 μL) to monitor the mixing process. The glycerol droplet is stirred by the rotation of JO-robot (without NIR laser irradiation). It shows that with the rotation of JO-robot, the fluorescence gradually diffuses to the whole glycerol droplet. However, the diffusion speed is slow. As can be seen from the normalized two-dimensional fluorescence distribution maps, it takes 30 minutes to stir to make the fluorescence intensity in the glycerol droplet uniform. In contrast, JO-robot can realize rapid and uniform mixing of glycerol droplet by photothermal stirring in just 4 minutes. Scale bar is 500 μm.

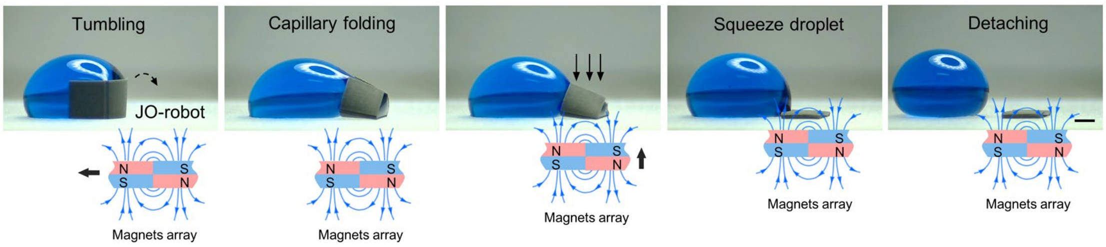

Supplementary Figure 28. Detachment of JO-robot with folding. After completing a series of tasks, JO-robot can separate from the droplet. As the magnets array moves to the left, the JO-robot tumbles to the right and wraps a small portion of the liquid under the capillary force. By increasing the magnetic field strength (magnets array close to JO-robot), the JO-robot squeezes the wrapped liquid back into the large droplet. And then the JO-robot detaches from the large droplet and tumbles away directionally. Scale bar is 1 mm.

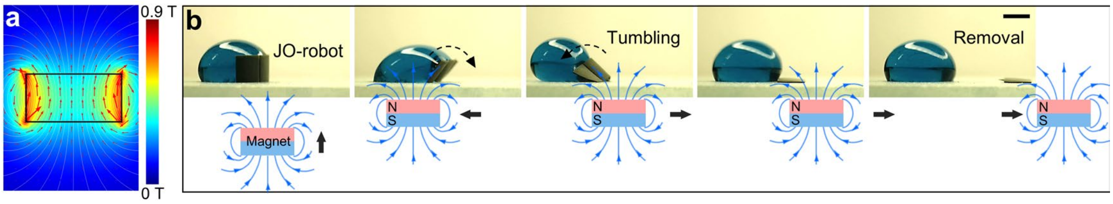

Supplementary Figure 29. Removal of JO-robot without folding. (a) The magnetic field simulation. A single neodymium-iron-boron (NdFeB) permanent magnet (20 mm × 20 mm × 10 mm) is used to remove the JO-robot without wrapping liquid. The N-pole and S-pole are distributed up and down along the magnet. The magnetic field strength at the edge of the magnet is larger than that in the middle. (b) Optical images of the JO-robot removal process. First, the magnet approaches the JO-robot from the bottom. When approaching, the center of the magnet is located at the lower left of the JO-robot. JO-robot tilts to the right under the action of strong magnetic torque, and the plates on both sides of the robot unfold. Then the magnet moves to the right. The JO-robot gradually tumbles to the left along the magnetic field direction and squeezes out the liquid between it and the substrate. As the magnet keeps moving to the right, the angle between the JO-robot and the substrate gradually decreases until JO-robot is completely fit to the substrate. At this time, the liquid is squeezed back into the large droplet. As the magnet moves to the right, the JO-robot gradually approaches the edge of the magnet, where the magnetic attraction becomes larger. The JO-robot is dragged away from the droplet by magnetic attraction, thus enabling controlled removal without folding, i.e., sliding out JO-robot using magnetic attraction. Scale bar is 2 mm.

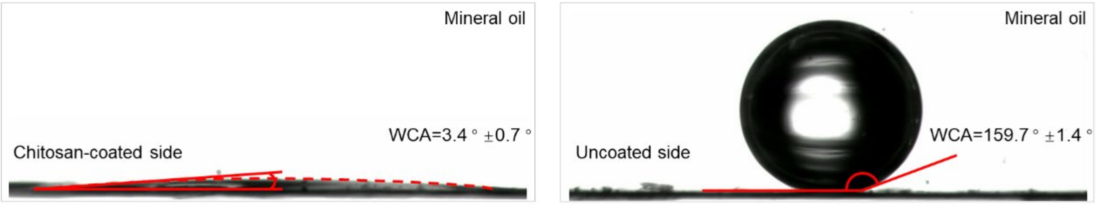

Supplementary Figure 30. Water contact angle of JO-robot in mineral oil. Before entering the mineral oil environment, the chitosan-coated side of the JO-robot is first pre-wetted with lysis buffer. The hydrophilicity of the surface (in air) makes the lysis buffer preferentially wet the surface, which hinders the infiltration of mineral oil. When the water droplet is added to the pre-wetted surface in mineral oil environment, the water merges with the liquid film in the structure, thus exhibiting the superhydrophilic property in the mineral oil. And the water contact angle (WCA) in mineral oil is 3.4° ± 0.7°. The uncoated side is oleophilic in air. When it is in the mineral oil environment, the mineral oil can preferentially wet the surface, which hinders the wetting of water. Therefore, the uncoated side is superhydrophobic in mineral oil (WCA=159.7° ± 1.4°).

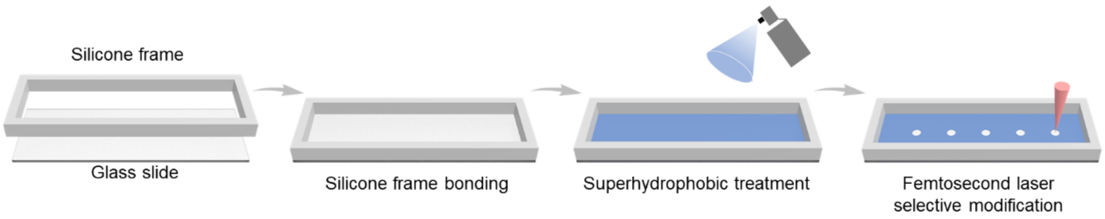

Supplementary Figure 31. Schematic diagram of the chip preparation process. The chip is mainly composed of a glass slide and a silicone frame. The silicone frame and the glass slide are joined together by plasma bonding. In order to make the reaction reagents stably fixed on the chip in the mineral oil environment, the surface of the chip needs to be modified. The commercial superhydrophobic spray is used to treat the chip to make the surface superhydrophobic. Several hydrophilic regions are then processed on the chip by femtosecond laser selective modification. The superhydrophobic material on the modified area is removed and the underlying hydrophilic glass is exposed. The surface of the glass becomes rough during the laser processing, which further enhances the hydrophilicity. Therefore, the processed regions are hydrophilic, while the unprocessed region is superhydrophobic. When the reagent is added to the hydrophilic area, it can preferentially wet the surface, which hinders the wetting of the mineral oil. Therefore, the reagent can be stably fixed on the chip in the mineral oil environment.

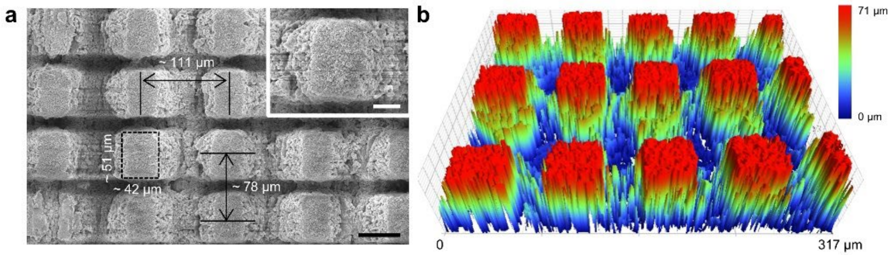

Supplementary Figure 32. SEM image and 3D surface topography image of the microcolumn array on the JO-robot surface. (a, b) In order to increase the nucleic acid binding area, the microcolumn array structure is prepared on the surface of JO-robot by femtosecond laser processing. (a) The size of the microcolumn is ~42 μm × ~51 μm. The spacing between the microcolumns is ~111 μm and ~78 μm, respectively. Scale bar is 50 μm. The inset shows an enlarged view of a single microcolumn. It can be seen that there are many micro pits on the side wall of the microcolumn. And there are many micro/nano particles on the top. These characteristics further increase the binding area of JO-robot to nucleic acid. Scale bar of the inset is 20 μm. (b) The 3D surface topography image of the microcolumn array on the JO-robot surface.

Supplementary Table 1. Comparison of droplet manipulation functions that can be achieved by magnetic additives-based droplet manipulation strategies (category I).

Function / Additive	Transport	Merging	Stirring (Active mixing)	Daughter droplet dispensing/ Splitting	On-demand daughter droplet release*	Heating
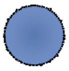 Hydrophobic magnetic powder[1]	✓	✗	✗	✗	✗	✗
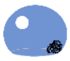 Hydrophilic magnetic particles[2]	✓	✓	✓	✓ Surface energy traps-assisted	✗ Pinned at designated regions	✗
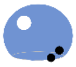 Steel beads[3]	✓	✓	✓	✓	✗ Entangled with the magnetic additives	✗
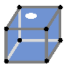 Printed solid frame[4]	✓	✓	✓	✓	✗ Random scatter	✗
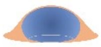 Ferrofluid[5]	✓	✓	✗	✓ Structural morphology -assisted	✗ Entangled with the magnetic additives	✗
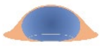 Ferrofluid[6]	✓	✓	✗	✗	✗	✗
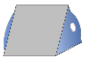 JO-robot (This work)	✓	✓	✓	✓	✓ On-demand release without magnetic additive	✓

Supplementary Table 2. Comparison of droplet manipulation functions that can be achieved by magneto-responsive surfaces-based droplet manipulation strategies (category II).

Function / Substrate	Transport	Merging	Stirring (Active mixing)	Daughter droplet dispensing/ Splitting	On-demand daughter droplet release*	Heating
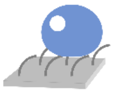 Microcolumns[7]	✓	×	×	×	×	×
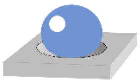 Microdimple[8]	✓	✓	✓	×	×	×
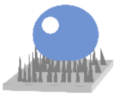 Microcilia[9]	✓	✓	✓	×	×	×
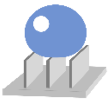 Microplate[10]	✓	✓	✓	×	×	×
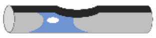 Microtube[11]	✓	×	×	×	×	×
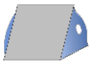 JO-robot (This work)	✓	✓	✓	✓	✓	✓

* Pure daughter droplet can be released on-demand at arbitrary positions on the open surface without any magnetic additives.

✓ denotes that the droplet manipulation functions can be realized by the strategies.

× denotes that the droplet manipulation functions are not mentioned in the literature.

Supplementary Table 3. Magnetic field information for different droplet manipulation functions.

Droplet manipulation function	Distance*	Required magnetic flux density**
Tumbling	<21 mm	>16.9±1.8 mT
Dispensing	8 mm~12 mm	81.5±5.7 mT~155.4±10.4 mT
Transportation	5 mm~16 mm	29.0±2.6 mT~227.4±13.9 mT
Releasing	<4 mm	>265.0±24.7 mT

* The distance between the working plane of the JO-robot and the upper surface of the magnets array. A continuous droplet manipulation process usually consists of the tumbling motion of JO-robot, the dispensing and transportation of droplet, so the distance should be kept between 8 mm and 12 mm to meet all the above manipulation requirements. When releasing the droplet, the distance between the magnets array and the working plane of JO-robot should be reduced (< 4 mm).

** For the tumbling of JO-robot, the dispensing and transportation of water droplet, the required magnetic flux density is described by the average value of the local maximum magnetic flux density in z-direction of the working space of JO-robot. The required magnetic flux density for droplet releasing is described by the horizontal magnetic flux density of the working space of JO-robot.

Supplementary Discussion 1. Magnetic excitation analysis of JO-robot

The periodic magnetic field used to excite the motion of JO-robot is generated by a permanent magnets array (the number of magnets is n, Supplementary Figure 33a).

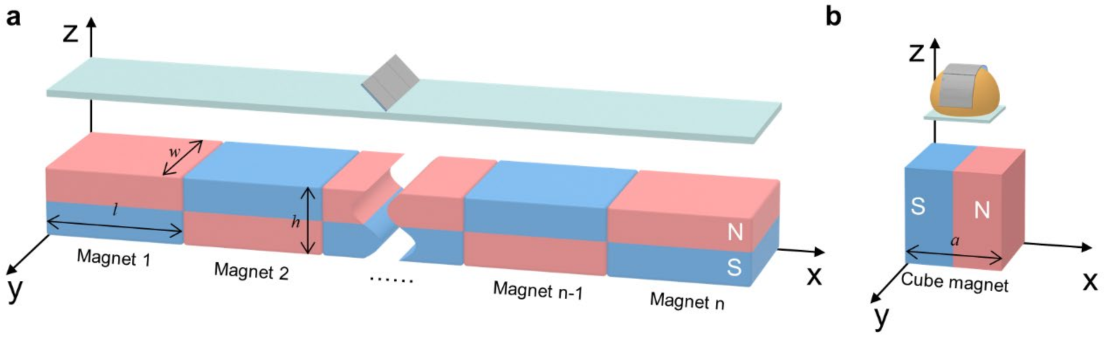

Supplementary Figure 33. Magnets for excitation of JO-robot. (a) Several permanent magnets with length of l, width of w and height of h are attracted end-to-end to form a magnets array (N-pole and S-pole are distributed up and down). (b) A cube permanent magnet with side length of a is used to drive the JO-robot to rotate. The N-pole and S-pole are distributed horizontally.

When the number of magnets is odd (n=2k-1, k=1, 2, …), the magnetic flux intensity **B** at an arbitrary point P (x, y, z) in the space out of the magnets can be described as[12]:

$$B_x = -\frac{\mu_0 J_S}{8\pi}[\Gamma((2k-1)l - x, y, z) + \Gamma((2k-1)l - x, w - y, z) - \Gamma(x, y, z) - \Gamma(x, w - y, z)] \quad (1)$$

$$B_y = -\frac{\mu_0 J_S}{8\pi}\left\{\sum_{k=1}[\Gamma(w-y, x+2(k-1)l, z) + \Gamma(w-y, (2k-1)l - x, z) - \Gamma(y, x+2(k-1)l, z) - \Gamma(y, (2k-1)l - x, z)] - \sum_{k=2}[\Gamma(w-y, x-(2k-3)l, z) + \Gamma(w-y, 2(k-1)l - x, z) - \Gamma(y, x-(2k-3)l, z) - \Gamma(y, 2(k-1)l - x, z)]\right\} \quad (2)$$

$$B_z = -\frac{\mu_0 J_S}{4\pi}[\phi(x,y,z) + \phi(y,x,z) + \phi(y,(2k-1)l - x,z) + \phi\big((2k-1)l - x,y,z\big) + \sum_{k=1}\big[\phi(x + 2(k-1)l, w - y, z) + \phi\big((2k-1)l - x, w - y, z\big) + \phi(w - y,(2k-1)l - x,z) + \phi(w - y, x + 2(k-1)l, z)\big] - \sum_{k=2}[\phi(x - (2k-3)l, w - y, z) + \phi(2(k-1)l - x, w - y, z) + \phi(w - y, 2(k-1)l - x, z) + \phi(w - y, x - (2k-3)l, z)] + \phi(y, 2kl - x, z) + \phi(2kl - x, y, z)] \tag{3}$$

When the number of magnets is even (n=2k, k=1, 2, …), the magnetic flux intensity **B** on an arbitrary point in the space *P* (x, y, z) can be described as[12]:

$$B_x = -\frac{\mu_0 J_S}{8\pi}[\Gamma(2kl - x,y,z) + \Gamma(2kl - x, w - y, z) + \Gamma(x,y,z) + \Gamma(x, w - y, z)] \tag{4}$$

$$B_y = -\frac{\mu_0 J_S}{8\pi}[\sum_{k=1}[\Gamma(w - y, x - (2k-1)l, z) + \Gamma(w - y, 2kl - x, z) - \Gamma(y, x - (2k-1)l, z) - \Gamma(y, 2kl - x, z)] - \sum_{k=2}[\Gamma(w - y, x - 2(k-1)l, z) + \Gamma(w - y,(2k-1)l - x, z) - \Gamma(y, x - 2(k-1)l, z) - \Gamma(y,(2k-1)l - x, z)]] \tag{5}$$

$$B_z = -\frac{\mu_0 J_S}{4\pi}[-\phi(x,y,z) - \phi(y,x,z) + \phi(y,2kl-x,z) + \phi(2kl-x,y,z) \tag{6}$$

$$+ \sum_{k=1}[\phi(x-(2k-1)l, w-y, z)$$

$$+ \phi(w-y, x-(2k-1)l, z) + \phi(2kl-x, w-y, z)$$
$$+ \phi(w-y, 2kl-x, z)]$$

$$- \sum_{k=2}[\phi(x-2(k-1)l, w-y, z)$$

$$+ \phi(w-y, (2k-1)l-x, z) + \phi\big((2k-1)l-x, w-y, z\big)$$

$$+ \phi(w-y, x-2(k-1)l, z)]]$$

in which

$$\Gamma(\gamma_1,\gamma_2,\gamma_3) = \ln\frac{\sqrt{\gamma_1^2+\gamma_2^2+(\gamma_3-Z_0)^2}-\gamma_2}{\sqrt{\gamma_1^2+\gamma_2^2+(\gamma_3-Z_0)^2}+\gamma_2}\Bigg|_{z_0=0}^{z_0=h} \tag{7}$$

$$\phi(\varphi_1,\varphi_2,\varphi_3) = \arctan\left[\frac{\varphi_1(\varphi_3-Z_0)}{\varphi_2\sqrt{\varphi_1^2+\varphi_2^2+(\varphi_3-Z_0)^2}}\right]\Bigg|_{z_0=0}^{z_0=h} \tag{8}$$

the *l*, *w* and *h* are the length, width and height of the magnet, respectively. B_x, B_y and B_z are the magnetic flux intensity along the x, y and z direction, respectively. μ_0 is the magnetic permeability of the vacuum. J_S is the magnetizing current density in the plane. The JO-robot can be subjected to the magnetic pulling force (**F**) and magnetic torque (**T**) when the magnets array moves along the *x* axis, which can be respectively calculated as[13]:

$$\mathbf{F} = V_m(\mathbf{M}\cdot\nabla)\mathbf{B}(x,y,z) \tag{9}$$

$$\mathbf{T} = V_m\mathbf{M}\times\mathbf{B}(x,y,z) \tag{10}$$

Where V_m is the volume of the JO-robot, **M** is the magnetization of JO-robot, **B**(*x, y, z*) is the magnetic flux intensity. The magnetic force attracts JO-robot to move toward the local maximum of the magnetic field. The magnetic torque aligns the internal magnetization **M** of JO-robot with the field **B**. That is, the carbonyl iron particles chains in the JO-robot are aligned with the magnetic field direction by the action of the magnetic torque. As shown in Supplementary Figure 34, the periodicity of the magnetic field makes JO-robot incline periodically. Therefore, when the magnets array moves along the *x* axis, JO-robot tumbles forward under the synergistic effect of magnetic force and magnetic torque. Similarly, JO-robot with water droplet can also move in a tumbling manner.

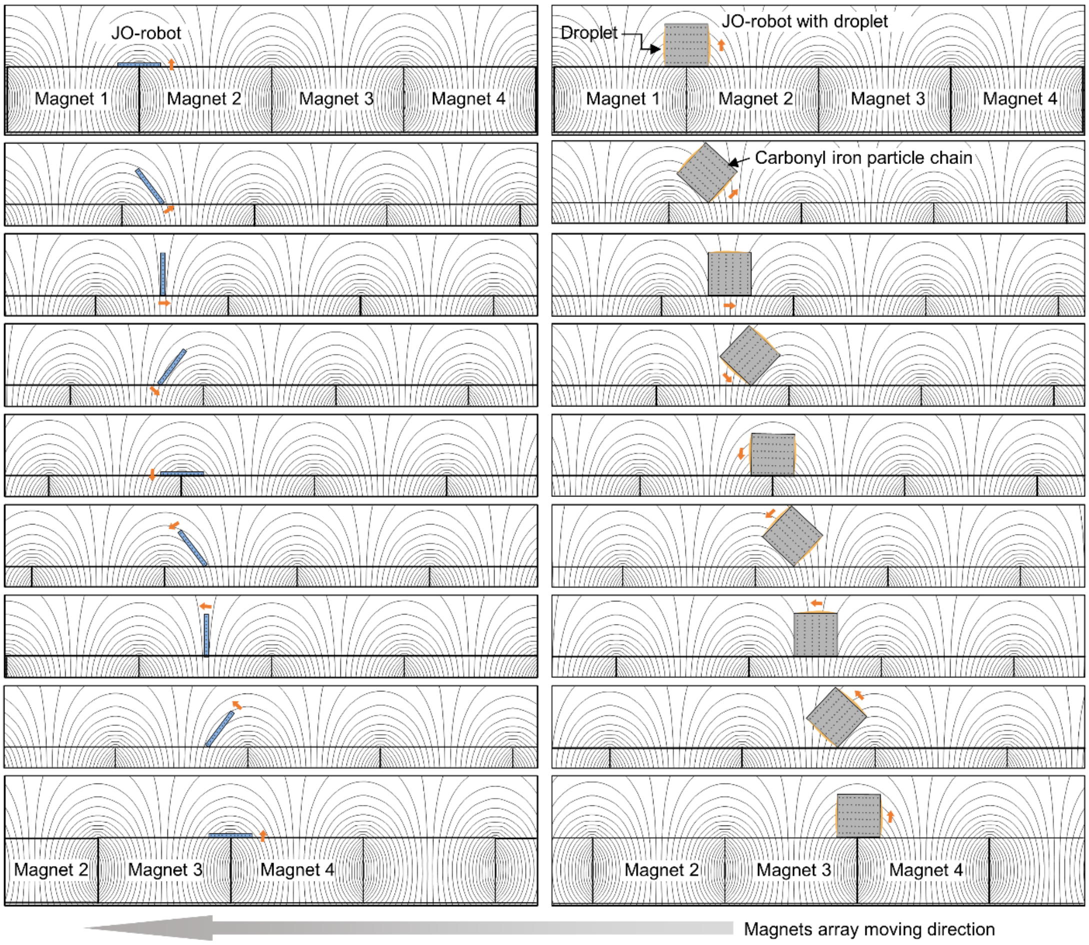

Supplementary Figure 34. Schematic illustration of the tumbling motion of JO-robot and JO-robot with droplet. When the periodic magnetic field moves along the *x* axis, the magnetic torque aligns the carbonyl iron particles chains in the JO-robot with the magnetic field direction. Since the magnetic field generated by the magnets array is periodic, JO-robot and JO-robot with droplet move in a tumbling manner.

The controllable rotation of JO-robot is realized by rotating the cube magnet (Supplementary Figure 33b., $w=h=l=a$). According to equation (1)-(3), the magnetic flux intensity $\mathbf{B}_c$ at an arbitrary point P (x, y, z) in the space out of the cube magnet can be described as:

$$B_x = -\frac{\mu_0 J_S}{8\pi}[\Gamma(a-x,y,z)+\Gamma(a-x,a-y,z)-\Gamma(x,y,z)-\Gamma(x,a-y,z)] \quad (11)$$

$$B_y = -\frac{\mu_0 J_S}{8\pi}[\Gamma(a-y,x,z)+\Gamma(a-y,a-x,z)-\Gamma(y,x,z)-\Gamma(y,a-x,z)] \quad (12)$$

$$B_z = -\frac{\mu_0 J_S}{4\pi}[\phi(x,y,z)+\phi(y,x,z)+\phi(y,a-x,z)+\phi(a-x,y,z)+\phi(x,a-y,z)+\phi(a-x,a-y,z)+\phi(a-y,a-x,z)+\phi(a-y,x,z)] \quad (13)$$

When the cube magnet rotates, the magnetic torque ($\mathbf{T}_m$) that JO-robot experienced under rotation magnetic field drives the JO-robot to rotate with the magnetic field (Supplementary Figure 35). And $\mathbf{T}_m$ can also be calculated by equation (10), i.e., $\mathbf{T}_m = V_m\mathbf{M}\times\mathbf{B}_c(x,y,z)$.

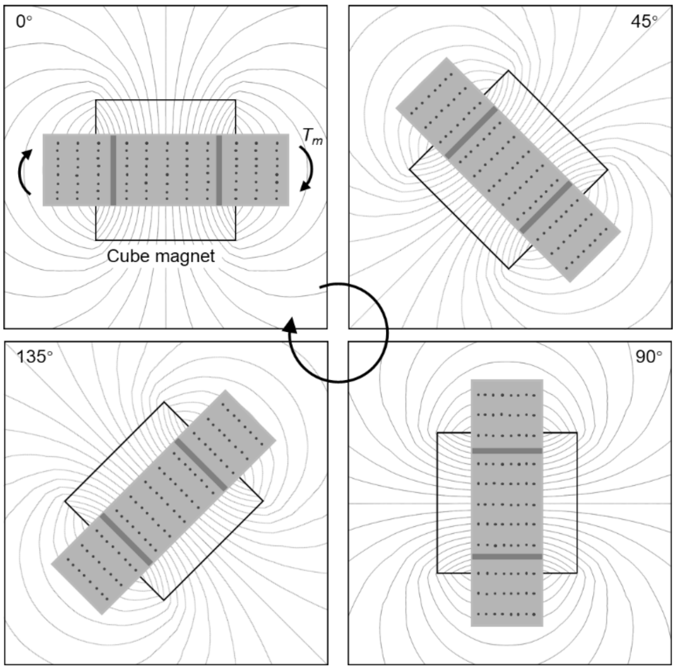

Supplementary Figure 35. Schematic illustration of the rotation of JO-robot driven by a cube permanent magnet (top view). Under the action of the rotating magnetic field, magnetic torque ($\mathbf{T}_m$) can be generated in JO-robot, which drives the carbonyl iron particles chains in the JO-robot to align with the direction of magnetic field, so as to rotate the JO-robot.

Supplementary Discussion 2. The total energy of the system

The total energy of JO-robot interacting with droplet can be expressed by:

$$E_t = E_k + E_\gamma + E_g + E_m \tag{14}$$

$$E_k = \sum_{i=1}^{n} \frac{1}{2} \int_{A_i} \mathbf{K}^T \mathbf{D} \mathbf{K}\, dA + \sum_{j=1}^{n-1} \frac{Ewh^3}{24(1-v^2)} \int_{S_j} k^2 ds \tag{15}$$

$$E_\gamma = \int_{A_{ls}} \gamma_{ls} dA + \int_{A_{sv}} \gamma_{sv} dA + \int_{A_{lv}} \gamma_{lv} dA \tag{16}$$

$$E_g = \int_{V_w} \rho_w gz dV + \int_{V_J} \rho_J gz dV \tag{17}$$

$$E_m = \int_{V_J} \mu_0 MH dV \tag{18}$$

where E_t is the total energy of the system, E_k are the strain energies of the plates bending and torsion and creases bending, E_γ are the interface energies, E_g are the gravitational energies of JO-robot and water droplet, and E_m is the magnetic energy. The i=1, 2, …, n is the number of the plates, **K** is the generalized strain matrix, **D** is the plate elasticity matrix, A_i is the area of the ith plate. j=1, 2, …, n-1 is the number of the creases, E is Young's modulus of the JO-robot, w is the width of the JO-robot, h is the thickness of the crease, v is the Poisson's ratio, s_j is the arc length of the jth crease, k is the curvature of the crease (Supplementary Figure 36). A_{ls}, A_{sv}, A_{lv} are the contact areas of the liquid-solid, solid-vapour and liquid-vapour, respectively. γ_{ls}, γ_{sv}, γ_{lv} are the surface tensions of the liquid-solid, solid-vapour and liquid-vapour interfaces. V_w, ρ_w are the volume and density of the water droplet, respectively. V_j, ρ_j are the volume and density of the JO-robot, respectively. g is the gravitational acceleration, z is the height coordinate. μ_0 is the magnetic permeability of the vacuum, M is the magnetization, H is the magnetic field.

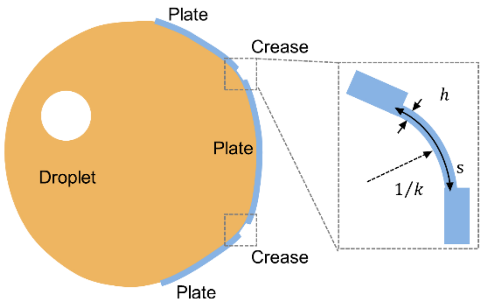

Supplementary Figure 36. Schematic illustration of the interaction between droplets and JO-robot. The JO-robot can be seen as three plates interconnected by two creases. The radius of curvature, arc length and thickness of the crease are *1/k*, *s* and *h*, respectively.

Supplementary Discussion 3. The relationship between the designed dimensional parameters and the volume of the dispensed and released droplets.

The volume of the dispensed daughter droplet can be managed by controlling the dimensional size of the JO-robot. The relationship between the designed dimensional parameters and the volume of the dispensed and released droplets is shown in Supplementary Figure 37. JO-robot can form a triangular prism after dispensing the droplet. The design volume of the dispensed droplet (V_d) can be approximately considered as the volume of the triangular prism: $V_d = 0.5ac(b^2\text{-}0.25c^2)^{1/2}$, where a is the width of the JO-robot, b is the distance between the crease and the short edge, c is the distance between the two creases (the creases are symmetrically distributed, Supplementary Figure 37a). However, due to the surface tension of water (water surface protrudes from the triangular prism) and the bending deformation of the robot caused by the capillary force, the actual dispensed droplet volume is larger than the design volume (Supplementary Figure 37c, 38a). When the droplet is squeezed out, the design volume of the residual liquid (V_r) can be approximately considered as the volume of the JO-robot folding gap: V_r=0.5at(c-b), where t is the thickness of the JO-robot (Supplementary Figure 37b). However, due to the elasticity of the JO-robot, the two plates cannot fit completely at the crease, resulting in residue at both creases, which makes the actual residue larger than the designed residue, that is to say, the volume of the actual squeezed droplet is slightly smaller than the design value (actual dispensed droplet volume minus design residual droplet volume, Supplementary Figure 37d, 38b).

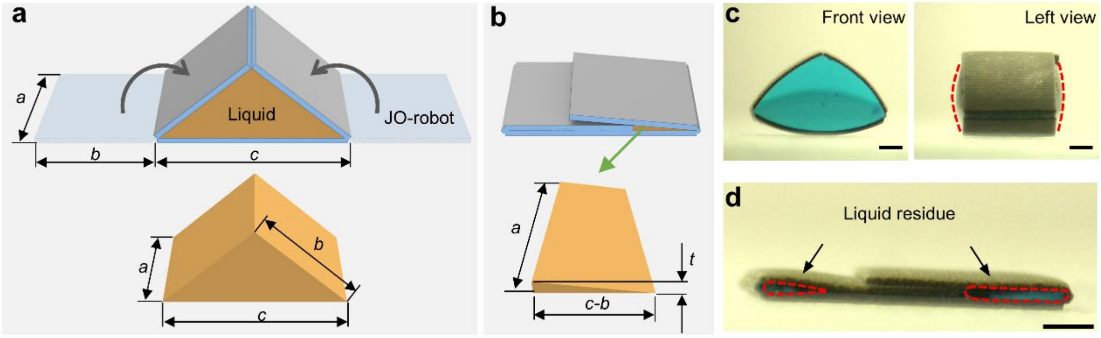

Supplementary Figure 37. Schematic diagram of the designed and the actual dispensed and residual liquid. (a, b) Relationship between the volume of dispensed/residual liquid and the size of JO-robot. (c, d) Optical images of actual dispensed droplet and residual liquid. Scale bars are 500 µm.

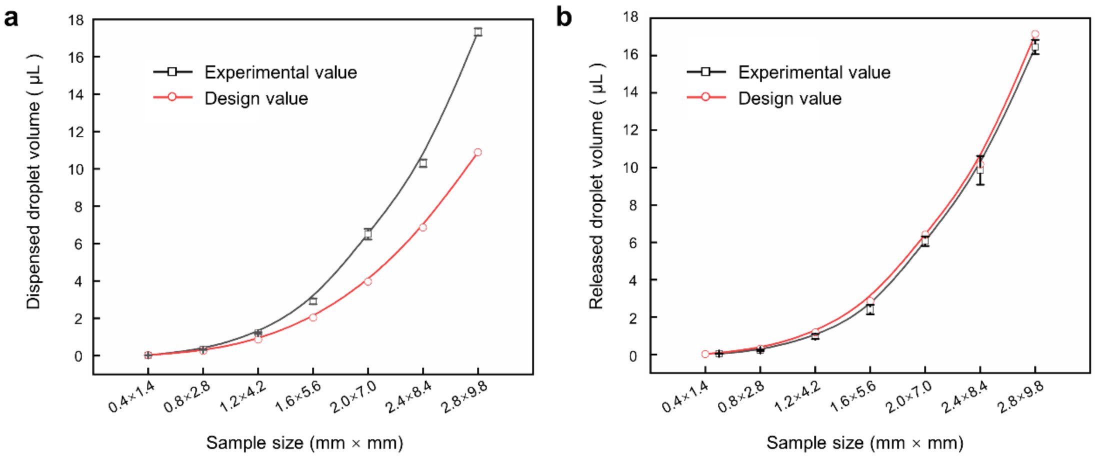

Supplementary Figure 38. Comparison of experimental and design values of droplets that (a) dispensed and (b) released by JO-robots with different sizes. The error bar represents the standard deviation of five independent measurements. Source data are provided as a Source Data file.

References

1. Zhao, Y., Xu, Z., Niu, H., Wang, X. & Lin, T. Magnetic liquid marbles: toward "lab in a droplet". *Adv. Funct. Mater.* **25**, 437-444 (2015).
2. Zhang, Y. & Wang, T. H. Full-range magnetic manipulation of droplets via surface energy traps enables complex bioassays. *Adv. Mater.* **25**, 2903-2908 (2013).
3. Li, A. et al. Programmable droplet manipulation by a magnetic-actuated robot. *Sci. Adv.* **6**, eaay5808 (2020).
4. Zhang, Y. *et al.* Magnetic-actuated "capillary container" for versatile three-dimensional fluid interface manipulation. *Sci. Adv.* **7**, eabi7498 (2021).
5. Zhang, J. *et al.* Wetting ridge assisted programmed magnetic actuation of droplets on ferrofluid-infused surface. *Nat. Commun.* **12**, 7136 (2021).
6. Nasirimarekani, V., Benito-Lopez, F. & Basabe-Desmonts, L. Tunable superparamagnetic ring (tSPRing) for droplet manipulation. *Adv. Funct. Mater.* **31**, 2100178 (2021).
7. Lin, Y. C. *et al.* Magnetically induced low adhesive direction of nano/micropillar arrays for microdroplet transport. *Adv. Funct. Mater.* **28**, 1800163 (2018).
8. Liu, H. *et al.* Magnetic actuation multifunctional platform combining microdroplets delivery and stirring. *ACS Appl. Mater. Interfaces* **11**, 47642-47648 (2019).
9. Demirors, A. F., Aykut, S., Ganzeboom, S., Meier, Y. A. & Poloni, E. Programmable droplet manipulation and wetting with soft magnetic carpets. *Proc. Natl. Acad. Sci. U.S.A.* **118**, e2111291118 (2021).
10. Jiang, S. *et al.* Three-dimensional multifunctional magnetically responsive liquid manipulator fabricated by femtosecond laser writing and soft transfer. *Nano Lett.* **20**, 7519-7529 (2020).
11. Lei, W. et al. High-speed transport of liquid droplets in magnetic tubular microactuators. *Sci. Adv.* **4**, eaau8767 (2018).
12. Gou, X.-f., Yang, Y., & Zheng, X.-j. Analytic expression of magnetic field distribution of rectangular permanent magnets. *Appl. Math. Mech.* **25**, 297-306 (2004)
13. Yang, X. et al. An agglutinate magnetic spray transforms inanimate objects into millirobots for biomedical applications. *Sci. Robot.* **5**, eabc8191 (2020).
